# Extensive range persistence in peripheral and interior refugia characterizes Pleistocene range dynamics in a widespread Alpine plant species (*Senecio carniolicus*, Asteraceae)

**DOI:** 10.1111/j.1365-294X.2012.05456.x

**Published:** 2012-03

**Authors:** Pedro Escobar García, Manuela Winkler, Ruth Flatscher, Michaela Sonnleitner, Jana KrejčíKová, Jan Suda, Karl HüLber, Gerald M Schneeweiss, Peter SchöNswetter

**Affiliations:** 1 Department of Systematic and Evolutionary Botany, University of ViennaRennweg 14, A-1030 Vienna, Austria; 2 Institute of Botany, Academy of Sciences of the Czech RepublicZámek 1, CZ-252 43 Průhonice, Czech Republic; 3 Department of Botany, Faculty of Science, Charles University in PragueBenátská 2, CZ-128 01 Prague, Czech Republic; 4 Vienna Institute for Nature Conservation & AnalysesGiessergasse 6/7, A-1090 Vienna, Austria; 5 Department of Conservation Biology, Vegetation Ecology and Landscape Ecology, University of ViennaRennweg 14, A-1030 Vienna, Austria; 6 Institute of Botany, University of InnsbruckSternwartestrasse 15, A-6020 Innsbruck, Austria

**Keywords:** AFLPs, Alps, geographic diffusion models, phylogeography, plastid sequences, Pleistocene refugia, range dynamics, *Senecio carniolicus*

## Abstract

Recent evidence suggests that survival of arctic-alpine organisms in peripheral or interior glacial refugia are not mutually exclusive and may both be involved in shaping an organism’s Pleistocene history, yet potentially at different time levels. Here, we test this hypothesis in a high-mountain plant (diploid lineage of *Senecio carniolicus*, Asteraceae) from the Eastern European Alps, in which patterns of morphological variation and current habitat requirements suggest survival in both types of refugia. To this end, we used AFLPs, nuclear and plastid DNA sequences and analysed them, among others, within a graph theoretic framework and using novel Bayesian methods of phylogeographic inference. On the basis of patterns of genetic diversity, occurrence of rare markers, distribution of distinct genetic lineages and patterns of range connectivity both interior refugia in the formerly strongly glaciated central Alps and peripheral refugia along the southern margin of the Alps were identified. The presence of refugia congruently inferred by markers resolving at different time levels suggests that these refugia acted as such throughout several glacial cycles. The high degree of range persistence together with gradual range expansion, which contrasts with the extent of range shifts implied for other Alpine species, is likely responsible for incipient lineage differentiation evident from the genetic data. Replacing a simplistic peripheral vs. interior refugia dualism by more complex models involving both types of refugia and considering different time levels will help identifying common phylogeographic patterns with respect to, for instance, location of refugia and colonization routes and elucidating their underlying genetic and/or ecological causes.

## Introduction

A central question of arctic-alpine phylogeography is concerned with Pleistocene range dynamics and the location of refugial areas (peripheral vs. interior refugia, so-called nunataks; reviewed in Stehlik 2000). Potentially governed by the inherent difficulty of providing convincing evidence for nunatak survival because of, for instance, extirpation of the likely small nunatak populations or genetic swamping by geographically close peripheral populations (Stehlik *et al.* 2001; Hewitt 2004), results of numerous phylogeographic studies within the last decades have diminished the importance of nunatak survival and identified peripheral refugia as the most relevant ones (Gabrielsen *et al.* 1997; Schönswetter *et al.* 2005). Thus, the discussion on peripheral vs. nunatak survival appears to have been settled in favour of the former (but see Holderegger *et al.* 2011). On the occasion of recent evidence for nunatak survival in arctic-alpine plants and animals (Lohse *et al.* 2011; Westergaard *et al.* 2011), it has been (re-)emphasized that nunatak and peripheral survival are not mutually exclusive and actually may both be involved in an organism’s Pleistocene history, yet potentially at different time levels (Schneeweiss & Schönswetter 2011).

The European Alps provide a suitable geographic model system for testing hypotheses on glacial survival of mountain species during the last glacial. On the basis of distributional (Merxmüller 1952, 1953, 1954; Pawłowski 1970) and molecular data (Paun *et al.* 2008; Burnier *et al.* 2009; Schneeweiss & Schönswetter 2010), peripheral refugia at the edges of the Alpine arc have been identified. In contrast, convincing evidence for nunatak survival in central parts of the Alps is much scarcer (Stehlik *et al.* 2002; Bettin *et al.* 2007; Parisod & Besnard 2007). Both results may, however, be misleading. Wide application (Schönswetter *et al.* 2002, 2003, 2004; Tribsch *et al.* 2002) of well-resolving, but rapidly homogenizing AFLP markers may cause underestimation of nunatak survival, especially in species with high colonizing capabilities that are particularly prone to genetic swamping (Gabrielsen *et al.* 1997; Schneeweiss & Schönswetter 2011). (In the context of AFLPs, homogenization refers to the rapid decay of signals of immigrant genotypes as a result of their repeated backcrossing with resident genotypes: Zhou *et al.* 2005). Geographically or phylogenetically restricted sampling (Stehlik *et al.* 2001; Bettin *et al.* 2007; Parisod & Besnard 2007) may result in overestimation of nunatak survival in case colonization from unstudied regions or interspecific gene flow from not sampled congeners remain unrecognized. Therefore, additional studies accounting for those potential pitfalls and using molecular markers resolving at different time levels are necessary for better assessment of modes of refugial survival in the Alps.

Identification of spatial genetic patterns, such as traces left by glacial refugia, remains an important objective of phylogeography, but yields only a snapshot of an organism’s history. Additional aspects of interest include, for instance, connectivity among different refugial areas or modes and directions of range expansions. Although such aspects may be tested using statistical phylogeographic approaches (Knowles & Maddison 2002; Knowles 2009), the sheer amount of plausible hypotheses that often need to be considered renders their application unfeasible except for simple settings (e.g. Spellman & Klicka 2006; Carstens & Richards 2007). Statistically rigorous methods for inferring phylogeographic history in a continuous landscape have been implemented in a maximum-likelihood framework (Lemmon & Lemmon 2008). More recently, this has been achieved in a Bayesian framework (Lemey *et al.* 2009, 2010) within the software beast (Drummond & Rambaut 2007), thus accommodating uncertainties in inferences of genealogy and of geographic diffusion. These methods allow establishing phylogeographic hypotheses with respect to range connectivity or ancestral locations in the absence of *a priori* knowledge, but to our knowledge, none of the Bayesian methods has been applied to plants yet.

**Fig. 1:**
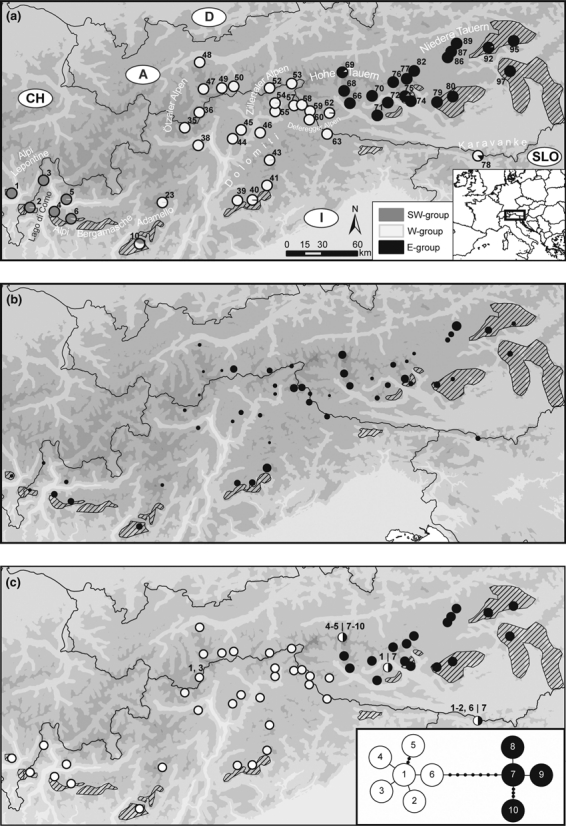
Physical map of the distribution of the analysed populations (a) and patterns of AFLP and ITS sequence variation in diploid *Senecio carniolicus*. (a) Phylogeographic grouping of populations according to Bayesian clustering analysis of AFLP phenotypes conducted with Structure. (b) Within-population rarity of AFLP markers (frequency-down-weighted marker values), its magnitude being proportional to dot size. (c) Distribution of ITS ribotypes, their relationships (inferred using statistical parsimony) shown in the insert; unless otherwise indicated, white and black dots represent ribotypes 1 and 7, respectively. In (a), populations are numbered as in Sonnleitner *et al.* (2010) and toponyms mentioned in the text are indicated. Hatched areas indicate potential glacial refugia on siliceous bedrock (modified from Schönswetter *et al.* 2005).

A good model system for addressing the complexity of Pleistocene range dynamics in alpine to subnival species is the diploid lineage of *Senecio carniolicus* (Asteraceae). Although commonly growing together, diploid and polyploid (tetraploids and hexaploids: Suda *et al.* 2007) cytotypes of *S. carniolicus* are reproductively isolated by different habitat requirements (Schönswetter *et al.* 2007; Hülber *et al.* 2009; Sonnleitner *et al.* 2010) as well as strong crossing barriers (Sonnleitner *et al.* 2010; M. Sonnleitner *et al.*, unpublished) and apparently constitute distinct and nonintermixing lineages. Therefore, it is justified to restrict ourselves here to the diploid cytotype. Several lines of evidence suggest that both peripheral and internal refugia may have played a role in its Pleistocene history. Diploid *S. carniolicus* occurs in essentially all peripheral refugia identified for silicicolous species in the Eastern Alps (Schönswetter *et al.* 2005; Thiel-Egenter *et al.* 2009; the locations of these refugia are shown in Fig. 1a). Furthermore, taxonomically acknowledged (as var. or subsp. *insubricus*) morphological differentiation between populations from the southwestern Eastern Alps and those elsewhere suggests (under a neutral model) sufficiently long and/or repeated isolation within the prominent and geographically stable refugium in the Southern Alps. On the other hand, diploid *S. carniolicus* grows in exposed, rocky habitats up to more than 3100 m a.s.l. (Hülber *et al.* 2009; Sonnleitner *et al.* 2010), rendering occurrence on steep south-exposed slopes, which likely served as microclimatically favourable habitats on nunataks (Parisod & Besnard 2007), a valid hypothesis. Additionally, nunatak survival has already been suggested for *S. halleri* (Bettin *et al.* 2007), a closely related endemic of the Western Alps with similar habitat requirements.

Here, we test the hypothesis that Pleistocene range dynamics of diploid *S. carniolicus* were influenced by survival in both peripheral and interior refugia, even if these potentially acted at different time levels. To this end, based on comprehensive geographic sampling and taking closely related congeners into account, we employ several different molecular markers: rapidly homogenizing, biparentally inherited AFLPs, which often are used to resolve intraspecific relationships because of their ability to provide phylogenetic signal in young or rapidly evolving study systems (Després *et al.* 2003; McKinnon *et al.* 2008); biparentally inherited nuclear ITS sequences, which often provide enough information for addressing evolutionary questions at the species level, including hybridization (Nieto Feliner & Rosselló 2007); and maternally inherited (for Asteraceae: Corriveau & Coleman 1988) plastid DNA sequences, which are less likely to be genetically swamped owing to their higher propensity of introgressing into immigrants (Currat *et al.* 2008) and whose slower mutation rates (Petit *et al.* 2005; Shaw *et al.* 2005; Borsch & Quandt 2009) allow tracing of patterns pre-dating the last glacial maximum. We analysed the molecular data, among others, within a graph theoretic framework and using novel Bayesian methods of phylogeographic inference. Our first aim is to identify putative refugia based on patterns of genetic diversity, occurrence of rare markers, and distribution of distinct genetic lineages (Taberlet 1998; Hewitt 1999, 2000; Paun *et al.* 2008; Provan & Bennett 2008), including testing hypotheses of previously defined peripheral refugia for silicicolous species (Schönswetter *et al.* 2005) via their correspondence with genetic groups; in case both peripheral and interior refugia are found, we additionally assess whether these acted as refugia at the same time levels or not. Our second aim is to infer relationships among different refugia as well as the (re)colonization directionality, specifically, whether there is a general eastward migration trend as expected from the distribution of the closest relatives of *S. carniolicus* (*S. halleri* and *S. incanus*) in the Western Alps.

## Materials and methods

### Study species

*Senecio carniolicus* (syn. *Jacobaea carniolica*; Pelser *et al.* 2006) is a common and abundant acidophilic species of the Eastern Alps and the Carpathians, where it grows in alpine grasslands, moraines and stable screes up to 3300 m a.s.l. (Reisigl & Pitschmann 1958). Within the Eastern Alps, *S. carniolicus* comprises three main cytotypes (diploids with 2*n* = 2*x* = 40, tetraploids with 2*n* = 4*x* = 80, hexaploids with 2*n* = 6*x* = 120; Suda *et al.* 2007; Sonnleitner *et al.* 2010). Diploids are reproductively isolated from polyploids by different habitat requirements (Sonnleitner *et al.* 2010) as well as strong crossing barriers (crossings of diploids with polyploids produce nearly no seeds, and these show reduced viability; M. Sonnleitner *et al.*, unpublished), and we only consider the diploid lineage here. Populations of *S. carniolicus* from the southwestern Eastern Alps have more deeply lobed and more densely hairy leaves, causing morphological resemblance to *S. incanus*, a closely related, but morphologically and genetically clearly distinct species (Hess *et al.* 1980; Pelser *et al.* 2003) from the Western Alps and the northern Apennines. These forms, initially described as *S. carniolicus* var. *insubricus* (Chenevard 1906), are no hybridogenic intermediates, but phylogenetically clearly belong to *S. carniolicus* (Pelser *et al.* 2003; Fig. S1).

### Plant material

From those populations analysed by Sonnleitner *et al.* (2010), where diploids were found, two to eight diploid individuals per population were included. No plant material was available from their populations 24 and 96 (depleted after flow cytometry), resulting in 52 studied populations (Table S1), which cover the entire distribution range of diploid *S. carniolicus*. This sampling also included several populations of *S. carniolicus* var. *insubricus* (pops. 1–6). As outgroups for some analyses, we included samples of all members of the *Incani* clade (Pelser *et al.* 2003, 2004), that is, *S. abrotanifolius*, *S. adonidifolius*, *S. boissieri*, *S. halleri*, *S. incanus*, *S. leucophyllus* and *S. personii* (Appendix S1, Table S2). Leaf material was collected and immediately stored in silica gel. Voucher specimens are deposited at the Institute of Botany, University of Vienna, Austria (WU, voucher numbers and collecting details are given in Sonnleitner *et al.* 2010).

### Molecular methods

Total genomic DNA was extracted from similar amounts of dried tissue (*c.* 10 mg) with the DNeasy 96 plant mini kit (Qiagen, Hilden, Germany) following the manufacturer’s protocol.

The AFLP procedure followed Vos *et al.* (1995) with the modifications described in Schönswetter *et al.* (2009). Initially, selective primers were screened using 16 primer combinations. The three final primer combinations for the selective PCR (fluorescent dye in brackets) were *Eco*RI (6-Fam)-ACA/*Mse*I-CAT, *Eco*RI (VIC)-AGG/*Mse*I-CTC, and *Eco*RI (NED)-ACA/*Mse*I-CAC. Purification and visualization of PCR products as well as scoring were carried out as described in Rebernig *et al.* (2010b). Six plants were extracted twice to test the reproducibility of AFLP fingerprinting (Bonin *et al.* 2004). Seven samples were used as replicates between PCR plates and were replicated more than twice, resulting in a total of 75 replicates. The error rate (Bonin *et al.* 2004) was calculated as the ratio of mismatches (scoring of 0 vs. 1) over phenotypic comparisons in AFLP profiles of replicated individuals. Nonreproducible fragments were excluded from the analyses.

PCR products of the nrDNA ITS region and the plastid spacers *trn*L-*rpl*32, *rps*16-*trn*K, *psb*D-*trn*T and *pet*L-*psb*E were obtained in reaction volumes of 13 μL including 4.5 μL REDTaq ReadyMix PCR mix (Sigma-Aldrich, Steinheim, Germany), 0.5 μL of 1 mg/mL bovine serum albumin (BSA; Promega, Madison, WI, USA), 0.5 μL 10 μm each of forward and reverse primers and about 25 ng of DNA suspended in 1 μL 1× TAE buffer. For ITS, primers and PCR conditions were as in Blöch *et al.* (2009). Plastid spacers were amplified using the primers of Shaw *et al.* (2007). Some samples from the outgroup taxa repeatedly failed to amplify and were amplified using newly designed internal primers listed in Table S3. PCR conditions were as follows: 95 °C for 1 min followed by 10 cycles each consisting of 30 s at 95 °C, 30 s at 47 °C, 1.5 min at 65 °C; 20 cycles each of 30 s at 95 °C, 30 s at 49 °C, 1.5 min at 65 °C; and a final elongation step of 8 min at 65 °C. PCR programs were run on GeneAmp PCR System 9700 thermocyclers (PE Applied Biosystems, Foster City, CA, USA). ITS PCR products from individuals in which length polymorphism made direct sequencing impossible were cloned using the pGEM-T-easy vector system and JM109 competent cells (Promega) following the manufacturer’s instructions. Inserts of 8–15 clones per individual were amplified using universal primers M13F(–47) and M13R(–48) and 1 μL of colony resuspended in 70 μL 1× TBE. PCR conditions were the same as described above except for annealing and elongation temperatures of 62 and 72 °C, respectively. PCR products were purified with *E. coli* Exonuclease I and FastAP Thermosensitive Alkaline Phosphatase (Fermentas, St. Leon-Rot, Germany) following the manufacturer’s instructions. Cycle sequencing reactions were performed using 5 μL of purified template and 1 μL BigDye Terminator (PE Applied Biosystems), then cleaned with Sephadex G-50 Fine (GE Healthcare Bio-Sciences, Uppsala, Sweden) and sequenced on an ABI 3770 DNA Analyzer (PE Applied Biosystems).

### Data analysis

#### AFLP data

All monomorphic fragments and the ones present in all but one individual of the diploid data set were removed from the data set to avoid biased parameter estimates (Bonin *et al.* 2004). Fragments present in only one diploid individual were only removed if they were singular with respect to a data set including all cytotypes of *S. carniolicus*.

Population structure was inferred employing a Bayesian clustering approach developed for dominant markers (Structure 2.2; Pritchard *et al.* 2000; Falush *et al.* 2007) with an admixture model with uncorrelated allele frequencies and recessive alleles. Ten replicate runs for each K (number of groups) ranging from 1 to 10 were carried out at the Bioportal of the University of Oslo (http://www.bioportal.uio.no/), using a burn-in of 10^5^ iterations followed by 10^6^ additional MCMC iterations. Similarity among results of different runs for the same K was calculated according to Nordborg *et al.* (2005) using Structure-sum-2009 (Ehrich 2006). We identified the optimal number of groups as the value of K where the likelihood reached a plateau; the results of replicate runs were identical, and no empty groups were encountered. Replicate runs of the best K were merged with Clumpp 1.1.1 (Jakobsson & Rosenberg 2007) using the ‘Full Search’-algorithm. The relative ‘cluster membership coefficients’ of all individuals were then averaged for each population. A principal coordinate analysis (PCoA) based on a matrix of Nei72 distances (Nei 1972) among individuals was calculated using the modules ‘Simgend’, ‘Dcenter’ and ‘Eigen’ from NTSYS-pc 2.2 (Rohlf 1997).

The frequency of rare markers as frequency-down-weighted marker values was calculated according to Schönswetter & Tribsch (2005) using AFLPdat (available from http://www2.uit.no/ikbViewer/page/ansatte/organisasjon/ansatte/person?p_document_id=41186&p_dimension_id=88165). These individual values were averaged to obtain values for the within-population rarity of markers (in the following termed ‘DW’).

The genetic covariance structure among sampled populations was modelled within a graph theoretic framework (Population Graphs: Dyer & Nason 2004) using PopGraphs (available from http://dyerlab.bio.vcu.edu/software/). A population network is constructed, where populations, which constitute the nodes, are connected by edges only if there is significant genetic covariance between the populations after removing the covariation each population has with the remaining populations in the data set. The distribution of shortest paths through the Population Graph creates an expectation of proportional distances for populations. If genetic covariance is spatially structured, the physical distances should be proportional to the genetic distances. If not, then the populations are either closer (compressed edges) or further apart (extended edges) than expected given the genetic distances (χ^2^ tests at α = 0.05 significance level). Extended edges may indicate long-distance dispersal, whereas compressed edges point to the presence of topological, historical and/or ecological sources of vicariance (Garrick *et al.* 2009).

Genetic covariance among geographic groups as defined for the phylogeographic analyses of the plastid sequence data (see next section) was also analysed using Population Graphs networks as just described. Stability of edges among geographic groups was assessed using a bootstrap approach with 200 bootstrap replicates. Pseudoreplicate data sets were generated using seqboot from the Phylip package (Felsenstein 1989) and analysed like the original data set. The proportion of replicates where a certain edge is found constitutes its bootstrap support.

#### Sequence data

Sequences were assembled using SeqMan II (DNAStar, Madison, WI, USA), manually edited and aligned with BioEdit 7.0.5.2 (Hall 1999). Within-population haplotype diversity was estimated using π, the mean number of pairwise differences (Tajima 1983), calculated with Arlequin 3.11 (Excoffier *et al.* 2005).

Nuclear ITS and plastid sequence data (the four regions concatenated under the assumption that the plastid genome is a single linkage group) were analysed using statistical parsimony as implemented in TCS (Clement *et al.* 2000) with the connection limit set to 95%. As gaps were treated as fifth character state, indels longer than 1 bp were recoded as single characters by reducing them to single base pair columns. Nested indels were coded as missing data. Mononucleotide repeats were removed owing to their high degree of homoplasy over larger geographic scales (Ingvarsson *et al.* 2003; Vachon & Freeland 2011). Prior to the TCS analysis, monophyly of the *S. carniolicus* ribotypes with respect to those of other species of the *Incani* clade was assessed via phylogenetic analyses using maximum parsimony in paup* 4.0b10 (Swofford 2003) and Bayesian inference in MrBayes 3.1.2 (Ronquist & Huelsenbeck 2003; see Appendix S1 for details).

Phylogeographic analyses of the plastid data set (comprising the four concatenated regions) were conducted in beast 1.6 (Drummond & Rambaut 2007). Model-fit of nucleotide substitution models was assessed via the Akaike Information Criterion (AIC) and the Bayesian Information Criterion (BIC) as implemented in jModelTest 0.1.1 (Posada 2008). Model uncertainty was considerable and the sets of models with cumulative AIC and BIC weights of at least 0.95 contained models differing in the number of substitution rates (3–6 vs. 1–3). Therefore, we finally used a HKY model with rate heterogeneity modelled by a gamma distribution (with six rate categories) to avoid over-parameterization. As prior for the transition-transversion ratio κ, we used a normal distribution with mean 0.41 (derived from the model-averaged value for this parameter determined via AIC and BIC) and a deliberately wide standard deviation of 0.4. Rate evolution was modelled in a strict clock framework, because a relaxed clock model had an only slightly better marginal log-likelihood and the coefficient of rate variation had its highest posterior density around zero (data not shown). Because of the lack of external calibrations, we used a strong prior on the substitution rate modelled with a lognormal distribution with a mean of 7.5 × 10^−3^ substitutions per site per million years, a standard deviation of 0.6 and an offset at 0.0, thus ensuring a modal value of the distribution around 4 × 10^−3^ substitutions per site per million years in line with previously suggested values (Yamane *et al.* 2003; Smith *et al.* 2008). As these values were obtained for groups phylogenetically very distant from Asteraceae, their applicability to *S. carniolicus* remains uncertain, and, consequently, the obtained age estimates should be interpreted with appropriate caution. As population model, we used the Bayesian skyline plot (Drummond *et al.* 2005) with a group interval *m* = 3. Stationarity of the Markov chain was determined using Tracer 1.4 (available from http://tree.bio.ed.ac.uk/software/tracer/).

Spatial distribution through time was inferred employing a discrete model of geographic diffusion. Under this model, rates of diffusion between *a priori* defined discrete locations are estimated using a continuous-time Markov chain model (Lemey *et al.* 2009). Starting from the unobserved location at the root of the tree derived from a uniform distribution over all sampled locations, dispersal proceeds along each branch according to this model and gives rise to the observed locations at the tips. The geospatial model may be reversible, that is, the diffusion rate between regions is identical in both directions, or nonreversible, that is, the diffusion rate in one direction can differ from that in the reverse direction. Application of a continuous model of geographic diffusion (Lemey *et al.* 2010), apparently appropriate for the largely continuous range of *S. carniolicus* (Fig. 1a), was not possible because of insufficient signal in the data evident from simple reconstruction of the priors during analyses (data not shown).

Although geographically distinct sampling localities constitute intuitive discrete geographic units, the enormous number of possible rate parameters renders their use in *S. carniolicus* impossible. To be able to delimit a manageable number of discrete geographic units in a largely continuous distribution range in a repeatable manner, we applied agglomerative hierarchical clustering to populations using the function agnes with default options (Euclidean distances and upgma clustering) in the package ‘cluster’ (Maechler *et al.* 2005) in R 2.8.0 (R Development Core Team 2008). Groups were delineated applying a certain geographic distance cut-off. As we wanted to address questions concerning peripheral vs. interior refugia, the value of this cut-off was chosen to result in sufficient geographic resolution (i.e. peripheral and central populations should be in different geographic units) with as few groups as possible. Eventually, using a geographic distance cut-off of about 40 km, 13 geographic groups were distinguished (Fig. S2).

The full diffusion matrix of the simpler reversible model for 13 groups contains 78 rate parameters and therefore is still grossly over-parameterized. Hence, we used Bayesian stochastic variable selection to reduce the number of nonzero rates. Following Lemey *et al.* (2009), we used a truncated Poisson prior with a mean of 0.693 (i.e. *ln*2) and an offset corresponding to the number of rates necessary to minimally connect all regions (i.e. number of regions minus 1), which puts 50% prior probability on the minimal rate configuration. Sensitivity to the choice of prior was assessed via rerunning the analysis with different prior means (0.1, 1, 2, 5, 10). For the nonreversible model (comprising 156 rate parameters), the Poisson prior was parameterized with a mean of 12 (i.e. number of regions minus 1) and an offset of 0 (P. Lemey, pers. comm.). Sensitivity was assessed via rerunning the analysis with different prior means as above. In all cases, we used equal expectations for all rates, that is, the prior on the diffusion rates is not informed by the geographic distances among geographic units. Two runs per parameterization, each for 2 × 10^8^ generations with sampling every 5000th generation, were conducted. As both runs converged on the same stationary distribution and effective sample size values safely exceeded 200, they were combined after removal of the first 10% of sampled generations as burn-in. All parameter estimates were based on these two runs combined (72 000 sampling points). To assess the robustness of the results with respect to different delimitation of discrete geographic units, a second analysis using eight regions (corresponding to a geographic distance of about 70 km; Fig. S2) was conducted, using the same prior parameterizations as above. Identification of well-supported rates was performed using the beast module RateIndicatorBF.

## Results

### AFLPs

The three AFLP primer combinations yielded 264 unambiguous polymorphic fragments after the removal of 16 nonreproducible and 31 invariable markers in 227 individuals. Six singular markers were retained because they occurred also in individuals of the data set comprising polyploid cytotypes, rendering it unlikely that these markers merely were artefacts. In the AFLP profiles from replicated samples, 294 differences were observed out of 23 925 phenotypic comparisons, resulting in an error rate of 1.23%.

The optimal number of groups in the Structure analysis was K = 3, comprising a southwestern, a western and an eastern group (Fig. 1a). The disjunct population 78 from the southeastern margin of the distribution area belonged to the western group. Only populations 1–6, 10, 75 and 78 showed admixture among the groups (using an arbitrary cut-off of 10% or more for the proportion of the minor group). The two-dimensional PCoA corroborated the pattern shown in the structure analysis (Fig. S3). The division between the eastern group and the other two groups was much more distinct with 50.4% of the variation explained, than the division between the western and the southwestern groups (7.4% of the variation explained).

DW values ranged from 0.91 in population 49 to 1.57 in population 89 (Table S1). DW values were highest in the western Niedere Tauern (population 89), in the southern Dolomiti (population 41), and in the central Alps (populations 57, 66, 68, 69, and 75; Fig. 1b).

Roughly one-fourth of the sampled populations remained unconnected in the Population Graphs network (Fig. S4). The covariance structure among populations of the southwestern group (populations 1–6) was characterized by compressed edges, indicating that population pairs were geographically closer to each other than expected given their genetic distances. The same was true for populations from the central Alps (populations 35, 47–49; 53 and 57; 69 and 71) and the southern Dolomiti (populations 40 and 43). Extended edges were predominantly oriented in west–east direction. Two edges connected the western and eastern group: a normal edge joined population 78 in the Karavanke and population 97 from the easternmost Central Alps, and the southern Dolomiti were connected with the Niedere Tauern by an extended edge (populations 40 and 87).

In the Population Graphs network of geographic regions, stable edges with a bootstrap support >50% occurred mainly among neighbouring regions (Fig. 3a), with the exception of edges connecting region R5 with R13 and R8 with R11. Two of the 13 regions (R1, R6) had no stable edges.

### ITS

GenBank accession numbers and ITS sequence statistics are provided in Tables S1 and S4, respectively. Ribotypes of *S. carniolicus* constituted a monophyletic clade (60% bootstrap support; 0.97 posterior probability; Fig. S1). Direct sequencing yielded unambiguous sequences in all cases except for populations 69, 72 and 78, which displayed within-individual ITS sequence length variation and required cloning. Chimeric ITS clones may be the result of PCR recombination and were removed. The statistical parsimony network of ITS ribotypes (Fig. 1c) recovered two groups separated by eight mutational steps. The circumscription of these groups, which were also recovered in the larger data set (Fig. S1), was congruent with the southwestern plus the western AFLP groups on the one hand and the eastern AFLP group on the other (Fig. 1a,c).

### Plastid DNA sequences

GenBank accession numbers and sequence statistics are given in Tables S1, S2 and S4, respectively. No inversions were detected. Phylogenetic analysis using beast revealed three clades: haplotype group 1 (posterior probability 0.96) comprising H1–H10, haplotype group 2 (posterior probability 1.00) consisting of H11 and haplotype group 3 (posterior probability 0.99) with H12–H35. Within haplotype group 3, a moderately supported (posterior probability 0.96) subgroup constituted by H20–H35 could be distinguished. The earliest differentiation among these three lineages (age given as median and its 95% highest posterior density interval) is estimated to have occurred 1.15 (0.28–2.34) mya, subsequent diversifications falling safely within the Pleistocene (data not shown).

**Fig. 2:**
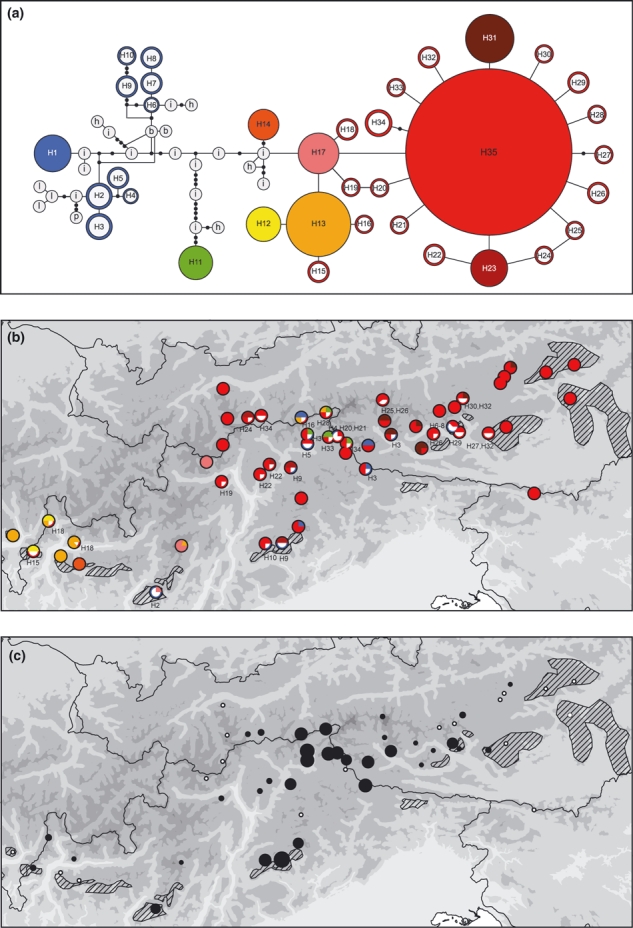
Patterns of plastid DNA variation in diploid *Senecio carniolicus*. (a) Statistical parsimony network of plastid haplotypes (blue, green and yellow to red colours correspond to haplotype groups 1, 2 and 3, respectively, identified by beast), a circle’s size being proportional to the square-root transformed frequency of the respective haplotype; haplotypes found in other species of the *Incani* clade are indicated by grey circles (b, *S. boissieri*; h, *S. halleri*; i, *S. incanus*; l, *S. leucophyllus*; p, *S. persoonii*) without indication of these haplotypes’ frequencies; unsampled haplotypes are represented by black dots. (b) Geographic distribution of plastid haplotypes; colours as in (a), haplotypes occurring in fewer than four individuals are marked with their number. (c) Within-population plastid haplotype diversity calculated as the mean number of pairwise differences (π), its magnitude being proportional to dot size (invariable populations indicated as white dots). Hatched areas in (b) and (c) as in Fig. 1.

A statistical parsimony network constituted of 35 plastid DNA haplotypes, and their geographic distribution are presented in Fig. 2a,b. The parsimony network of plastid DNA haplotypes revealed strong reciprocal differentiation among haplotypes pertaining to haplotype groups 1 and 3. The mean number of pairwise differences (π) calculated for sets of haplotypes of haplotype groups 1 and 3 amounted to 5.99 and 1.23, respectively (haplotype group 2 contains a single haplotype). The most frequent haplotype was H35 found in 53% of the investigated individuals throughout the distribution range except for its western third (Fig. 2b). Twelve further haplotypes (H17, H20, H21, H23, H25, H26, H28–H33) differed only in a single mutational step from H35 (together with H35 encompassing 71% of the investigated individuals). Most divergent were H1–H11, which were separated by at least 10 mutational steps from the other haplotypes. These haplotypes were present in 11% of the sampled individuals (Fig. 2b). Most outgroup haplotypes were similar to haplotypes H1–H11, but three haplotypes present in *S. incanus* and *S. halleri* were close to haplotypes H14 and H17 of haplotype group 3. No outgroup haplotypes were shared with the ingroup (Fig. 2a).

The mean number of pairwise differences within populations ranged from 0.0 (in 19 populations throughout the range) to 10.0 (population 40). The most diverse regions were the southern Dolomiti (populations 39–41) and the central Alps (populations 52–55, 57, 58, and 63; Fig. 2c, Table S1).

Qualitative inferences of geographic diffusion using a reversible discrete model were robust against prior parameterizations. Differences were of quantitative nature, as Bayes Factors (BF) usually increased (up to fourfold) with increasing prior means (Table S5a), which might cause BFs for some rates to eventually pass the applied threshold of 3 (the number of supported rates increased from 11 to 16 with prior means of 0.1 and 10, respectively: Table S5a). The composition of the set of rates congruently supported under all prior parameterizations essentially did not change and in two of the three cases, where rates showed decreasing BFs with increasing prior means, BFs dropped down to 2.7 (Table S5a). Qualitatively similar results were obtained with eight regions, even if trends in changes of BFs with changes of prior means were less clear (Table S5b).

In contrast, inferences using a nonreversible discrete model were highly sensitive to prior parameterizations. Specifically, the number of rates supported by BF ≥3 increased with *decreasing* prior mean from 25 to 133 rates (i.e. from 16% to 85% of possible rates) with prior means of 12 and 0.1, respectively (Fig. S5a). In the case of eight delimited regions, a similar increase was found from 13 to 48 rates (i.e. from 23% to 86% of possible rates) with prior means of 10 and 0.1, respectively (Fig. S5b). Furthermore, BFs of some rates increased enormously with decreasing means (Table S6); for instance that for R1→R2 increased more than 90-fold (Fig. S5a) compared to more than 400-fold in case of eight delimited regions (Fig. S5b). Finally, with decreasing prior means some results concerning rate support became dubious. Most conspicuously, rates involving the region hosting the single population in the southeastern Alps (pop. 78: R13) became frequently supported with decreasing prior means (2 vs. 23 of 24 possible rates with prior means of 12 vs. 0.1, respectively: Fig. S5a), although it harboured only a single widespread haplotype. Similar patterns were observed in case of eight delimited regions (1 vs. 11 of 14 possible rates with prior means of 10 vs. 0.1, respectively: Fig. S5b). Reasons for this odd behaviour may include convergence issues when using lower Poisson prior means (P. Lemey, pers. comm.), but a proper assessment would require extensive simulation studies going beyond the scope of this study. Consequently, results from the nonreversible model will be interpreted with caution taking into account the results from the more robust reversible model.

**Fig. 3:**
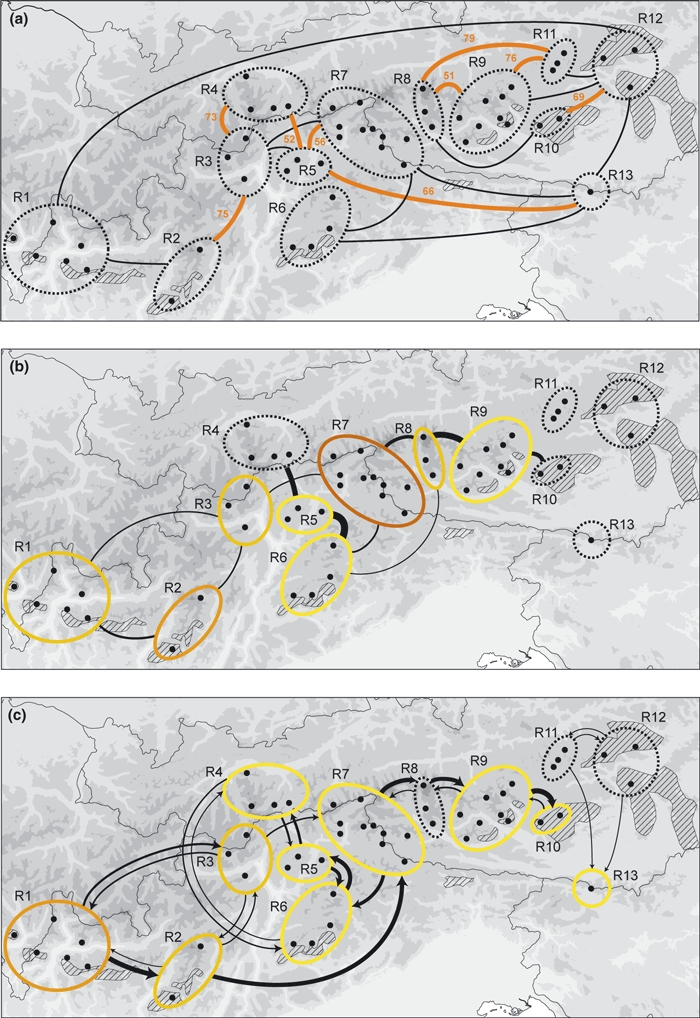
Range connectivity among discrete geographic regions (their designations R1–R13 are indicated) in diploid *Senecio carniolicus*. (a) Population Graphs network illustrating the genetic covariance structure among regions based on AFLP fingerprints; connections between regions indicated in orange are stable edges (with a bootstrap support >50% given at the edges), other edges are drawn in black. (b–c) Range connectivity inferred from plastid sequence data using (b) reversible and (c) nonreversible models of geographic diffusion between discrete geographic regions, the thickness of the connections being proportional to their support by Bayes Factors (see text for details); decreasing posterior probability (shown in four classes from 0.05 to 0.19) of a region to be the ancestral area is indicated by increasingly lighter colours. Hatched areas in (a) to (c) as in Fig. 1.

The reversible model identified 11 significant connections mostly between adjacent regions (Fig. 3b). The three easternmost regions remained unconnected (i.e. received BF support <3). The number of significant connections inferred from the nonreversible model was higher (five unidirectional and ten bidirectional ones: Fig. 3c), but this set included all rates identified with the reversible model except one (southern Dolomiti to central Hohe Tauern: R6→R8). The three easternmost regions were connected among each other, albeit only weakly supported, but remained unconnected from the remaining regions. In case of asymmetrical rates, eastward rates were better supported than westward ones. This is also the case for the connection from the southern Adamello region to the central Alps (R2→R7), whose support under the reversible model (2.99) is slightly below the applied threshold. A well-supported westward direction was inferred from the western Hohe Tauern and Zillertaler Alpen to the southern Dolomiti (R7→R6). Whereas the overall eastward directionality was insensitive to the number of delimited regions, the northward directionality weakly suggested for the central part (Fig. 3c) disappeared and was actually reversed with fewer regions [i.e. R(3–5)→R6 obtained higher support than the reverse rate: Fig. S5b].

Whereas under the reversible model the ancestral location was inferred to be in the central Alps (western Hohe Tauern and Zillertaler Alpen), under the nonreversible model it was situated in the southwestern region (Alpi Lepontine and Alpi Bergamasche). However, in both cases support was low (posterior probabilities <0.2, BF for single regions 0.35–2.8). The sets of regions with cumulative posterior probabilities of at least 0.8 included 8–10 regions (reversible and nonreversible model, respectively), only the two northeastern regions being congruently excluded.

## Discussion

Against previous, often confrontational assertions, glacial survival in peripheral refugia (massifs de refuge) does not preclude survival in interior refugia (nunataks), and both may contribute to shaping Pleistocene range dynamics of alpine biota (Stehlik *et al.* 2002; Lohse *et al.* 2011). This is also the case for diploid *Senecio carniolicus*, where several peripheral and interior refugia have been identified based on increased frequency of rare AFLP markers (Fig. 1b), elevated haplotype diversity and the distribution patterns of the oldest and hence most divergent haplotype lineages (Fig. 2). Suggested refugial areas are both along the formerly not or only weakly glaciated southern margin of the Alps (Alpi Bergamasche, southern Adamello and southern Dolomiti), where major refugia for silicicolous plants have been proposed before (Schönswetter *et al.* 2005), and within formerly strongly glaciated central parts of the cytotype’s range (Deferegger and Zillertaler Alpen). Remarkably, there is no evidence for survival of *S. carniolicus* in the easternmost Alps, which acted as a refugium for most silicicolous species investigated so far (e.g. *Saponaria pumila* and *Androsace wulfeniana*: Tribsch *et al.* 2002; Schönswetter *et al.* 2003). Instead, low frequency of rare AFLP markers (Fig. 1b), the near exclusive presence of the most widespread haplotype H35 (Fig. 2b) and inferred range expansions (Fig. 3) suggest recent, possibly postglacial, eastward range expansion into this area. The presence of refugia congruently inferred by markers resolving at different time levels suggests that these refugia acted as such throughout several glacial cycles and that diploid *S. carniolicus* responded to the Quaternary climatic oscillations in a cyclic manner (Rebernig *et al.* 2010a; Stewart *et al.* 2010).

An unexpectedly high degree of range persistence is evident not only from the presence of several refugia and their stability over time, but is also well reflected in patterns of range connectivity (Fig. 3) as usually only immediately adjacent areas are significantly connected. (It is worth emphasizing that neither PopGraphs nor beast, as used here, take distances between the geographic units into account; consequently any connections are deduced solely from the genetic data.) This pattern suggests that any range expansion occurred mostly gradually to nearby ranges; this also involves adjacent refugia (Fig. 3c). If sufficiently slow, this mode of (re-)colonization may be responsible for the preservation of the signal for interior refugia in the AFLP data, because populations in these refugia are particularly prone to genetic swamping because of their likely small population sizes (Stehlik 2000). Likewise, prolonged isolation in refugia and nonsaltatory range shifts are conducive for lineage differentiation, as is evident from the presence of a western and an eastern genetic group (AFLP and nuclear ITS data: Fig. 1a,c and Figs S1 and S3). The distribution areas of these groups nearly abut in the Hohe Tauern region, which roughly coincides with genetic discontinuities in other alpine taxa (Schönswetter *et al.* 2002, 2004, 2005; Thiel-Egenter *et al.* 2009; Fig. 1a). Only a few western populations of the eastern group (pops. 69, 72) show signs of admixture (Fig. 1c, Table S1) in line with low levels of gene flow across this contact zone. This main genetic split into a western and an eastern group does not coincide with the morphology-based taxonomic differentiation of *S. carniolicus* var. *insubricus* (i.e. the southwestern populations within the western group, which are also, but less strongly genetically differentiated: Fig. 1a and Fig. S3) from the nominal variety. However, a morphological re-evaluation of diploid *S. carniolicus* indicates that var. *insubricus* is morphologically similar to the remaining populations of the western lineage, but clearly divergent from the eastern group (Flatscher 2010). Finally, slow range shifts conferring potentially still incomplete range filling (Svenning *et al.* 2008) may also be responsible for the lack of diploid *S. carniolicus* in the ecologically suitable northwesternmost central Alps, although alternative hypotheses, such as niche pre-emption by the here abundant hexaploid cytotype, cannot be ruled out and require further studies.

Range persistence, however, does not imply range stasis, and both long-distance dispersal and range expansion contributed to the range dynamics of diploid *S. carniolicus*. This is in line with the presence of traits conducive to dispersal over longer distances exhibited by the diaspores, including low terminal velocities (determined on most likely hexaploid *S. carniolicus*: Tackenberg & Stöcklin 2008). Evidence for long-distance dispersal is most prominently found in the disjunct population 78 from the southeastern Alps (Fig. 1a). Although this population is spatially isolated, it displays unambiguous traces of admixture between the western and the eastern groups (Fig. 1a,c; Table S1; plastid data are not informative, because the population is fixed for the widespread haplotype H35: Fig. 2b). This is also reflected in the Population Graph analysis both at the level of populations (genetic covariance with pop. 57 from the western and pop. 97 from the eastern group: Fig. S4) and at the level of geographic regions (genetic covariance with the central and the easternmost Alps: Fig. 3a).

Unambiguous evidence for range expansion is found for the eastern half of the distribution area of diploid *S. carniolicus*, which likely has been (re-)colonized from the interior refugium around Deferegger and Zillertaler Alpen. This strictly eastward (re-)colonization is supported by the lack of putative refugial areas in the easternmost Alps (see above), the restriction of the most ancestral plastid DNA haplotypes (i.e. haplotype groups 1 and 2 that intermix with haplotypes found in the outgroup species) to the central parts of the entire distribution range (around western Deferegger and Zillertaler Alpen: Fig. 2), and the asymmetry in favour of eastward diffusion rates (Fig. 3c). Range expansions into northwestern regions, such as the Ötztaler Alpen, probably involved source areas corresponding to interior as well as peripheral refugia at the southern and southwestern part of the distribution range (Fig. 3c). Although (re-)colonization within the Alps from different refugia has been identified before (e.g. Schönswetter *et al.* 2005; Parisod & Besnard 2007), only the application of spatially explicit methods, as used here, allows a more detailed characterization of the (re-)colonization routes.

A general eastward directionality is expected from the distribution of *S. carniolicus*’ closest relatives from the *Incani* clade (Pelser *et al.* 2003). These are distributed throughout the western half of the southern European mountain systems from the Sierra Nevada and the Cordillera Cantabrica (*S. boissieri*) over the Pyrenees and the Massif Central (*S. leucophyllus*) to the Western Alps (*S. halleri*, *S. incanus*, *S. persoonii*), the Eastern Alpine and Carpathian *S. carniolicus* being the easternmost representative of the group. This hypothesis finds support in the nonreversible discrete diffusion model, where the most likely ancestral location is inferred in the southwesternmost portion of the distribution area (Alpi Bergamasche and Alpi Lepontine) and eastward diffusion rates generally predominate (Fig. 3c). This contrasts with westward directionality out from an ancestral location in the central part of the distribution range indirectly suggested by the location of the most likely ancestral area inferred with the reversible discrete diffusion model (Fig. 3b). The reasons for this contradiction are unclear, but the fact that the most likely ancestral area (Fig. 3b) harbours many of the ancestral haplotypes (Fig. 2) suggests an influence of the distribution of character states (i.e. geographic regions) on a genealogy on ancestral area reconstruction under the reversible model. The current restriction of ancestral haplotypes to central parts of the distribution area (Fig. 2) might be due to long-lasting isolation of populations within interior refugia. Such isolation may delay the possibilities of genetic replacement by immigrants from the periphery (similar to island populations: Hargreaves *et al.* 2010), thus assisting retention of ancestral haplotypes beyond the high propensity of organellar genomes to introgress into invading genomes (Currat *et al.* 2008). An alternative explanation for the presence of haplotype groups 1 and 2 in diploid *S. carniolicus* might be hybridization with closely related congeners. This is, however, unlikely based on geographic considerations (populations of *S. carniolicus* harbouring haplotype groups 1 and 2 are not geographically close to the potential source species *S. incanus* or *S. halleri*), rendering incomplete lineage sorting, possibly dating back to the late Pliocene, the more plausible hypothesis.

Using spatially explicit phylogeographic methods including novel Bayesian approaches has greatly advanced our understanding of Pleistocene range dynamics in a widespread Alpine plant species. Specifically, range dynamics of diploid *S. carniolicus* are characterized by remarkable range persistence in both peripheral (massifs de refuge) and interior refugia (nunataks), which likely acted as such recurrently through several glaciations cycles, and by independent range expansions out from these refugia in a manner determined mainly by geographic adjacency. This study emphasizes that our understanding of Pleistocene range dynamics even in a comparatively well-studied region like the Alps is still limited, negatively affecting identification of the most relevant patterns and processes. Future comparative studies will be necessary not only to identify common phylogeographic patterns with respect to, for instance, location of refugia and colonization routes (Carstens *et al.* 2004; Victoriano *et al.* 2008), but also to elucidate their underlying genetic and/or ecological causes (Álvarez *et al.* 2009).

## Data accessibility

DNA sequences have been deposited in GenBank under accession nos FR796701–FR797793 and nos HE614296–HE614583. Details regarding individual samples are available in Tables S1 and S2. Phylogenetic data (data matrices of ITS sequences and of coded plastid sequences, phylogenetic tree based on ITS sequences) are available at TreeBase (http://purl.org/phylo/treebase/phylows/study/TB2:S12125). AFLP data matrix, alignment of uncoded plastid sequences and exemplary beast input files are available at Dryad: doi: 10.5061/dryad.6v89fj76.

## Supporting information

**Appendix S1** Details of the phylogeneticanalyses of the *Incani* clade.

**Fig. S1** Phylogenetic relationships of *Senecio carniolicus* and related species (i.e., the *Incani* clade).

**Fig. S2** Population dendrogram based on agglomerative hierarchical clustering using geographic distances.

**Fig. S3** Principal Coordinate Analysis of AFLP data.

**Fig. S4** Population graphs network of AFLP data at the population level.

**Fig. S5** Bayes Factor support for diffusion rates in the discrete non-reversible geographic diffusion model under different parameterizations.

**Table S1** Population numbers, sampling locations and their coordinates, number of analysed individuals, population diversity measures, ITS ribotypes, plastid haplotypes, and GenBank accession numbers

**Table S2** Sampling localities and their coordinates as well as GenBank accession numbers of four plastid regions for outgroup species of the *Incani* clade

**Table S3** Newly designed internal primers for three plastid spacer regions

**Table S4** Sequence statistics

**Table S5** Bayes Factor support for geographic diffusion rates under the reversible discrete model

**Table S6** Bayes Factor support for geographic diffusion rates under the non-reversible discrete model
