# Supplementary material for: Extensive range persistence in peripheral and interior refugia characterizes Pleistocene range dynamics in a widespread Alpine plant species (Senecio carniolicus, Asteraceae)
Source: Mol Ecol. 2012 Mar;21(5):1255–70. doi: 10.1111/j.1365-294X.2012.05456.x (PMC3306793; doi:10.1111/j.1365-294X.2012.05456.x)
Supplement: Supplementary file 1 [file mec0021-1255-SD1.pdf]

Supplementary Material

Appendix 1 Details of the phylogenetic analyses of ITS sequences of the *Incani*-clade.
A data set of ITS sequences including one representative of each *S. carniolicus* population (Table 1) as well as 1–2 samples of other species of the *Incani*-clade (for details on sampling locations and their coordinates, voucher information, and on GenBank accession numbers, see table below) was constructed. For the maximum parsimony analyses, heuristic searches were conducted employing 1000 random sequence addition replicates and TBR branch swapping. Branch support was assessed by bootstrapping with 1000 pseudo-replicates, each with 10 random sequence addition replicates and TBR branch swapping saving 100 trees per replicate. The best-fit substitution model, determined via hierarchical likelihood ratio tests and the AIC as implemented in ModelTest 3.6 (Posada & Crandall 1998), was F81+I+G. For Bayesian inference, four runs with one cold and three heated chains each were run for 5 x 10^6 generations, sampling every 1000th generation. The first 25% of trees were discarded as burn-in, after which tree likelihoods and model parameters had reached stationarity. Clade posterior probabilities were calculated computing a 50% majority-rule consensus tree of the remaining 15,000 trees.

**Taxon**	**Voucher Information**	**Coordinates**	**Accession No.**
Senecio abrotanifolius L. 1	Croatia, Zadar, Velebit	N 44°31'45"<br>E 15°12'51"	HE585514
Senecio abrotanifolius L. 2	Italy, Sondrio, Bernina	N 46°11'40"<br>E 9°33'50"	HE585515
Senecio adonidifolius Loisel.	France, Pyrenées-Orientales,<br>East Pyrenees	N 42°29'<br>E 2°19'	HE585517
Senecio boissieri DC.	Spain, Granada, Mulhacén	N 37°03'14"<br>W 03°01'33"	HE585519
Senecio halleri Dandy	Italy, Aosta, Col d'Olen	N 45°51'48"<br>E 7°51'03"	HE585521
Senecio incanus L. 1	Switzerland, Valais,<br>Simplonpass	N 46°14'45"<br>E 08°01'30"	HE585522
Senecio incanus L. 2	Switzerland, Hérens,<br>Montagne d'Arolla	N 46°00'57"<br>E 07°26'15"	HE586331
Senecio leucophyllus DC.	France, Pyrénées Orientales,<br>Plas de Cady	N 42°30'30"<br>E 2°26'	HE585524
Senecio persoonii De Not.	Italy, Cuneo, Viozene	N 44°08'45"<br>E 7°47'05"	HE585526

Reference

Posada D, Crandall KA (1998) MODELTEST: testing the model of DNA substitution. *Bioinformatics*, **14**, 817–818.

Fig. S1 Phylogenetic relationships of *Senecio carniolicus* and related species inferred from Bayesian analysis of nuclear ITS sequences (the outgroup *S. jacobaea* [GU818563-67] not shown). Numbers above branches are branch lengths (number of steps), those below branches are maximum parsimony bootstrap values / Bayesian posterior probabilities.

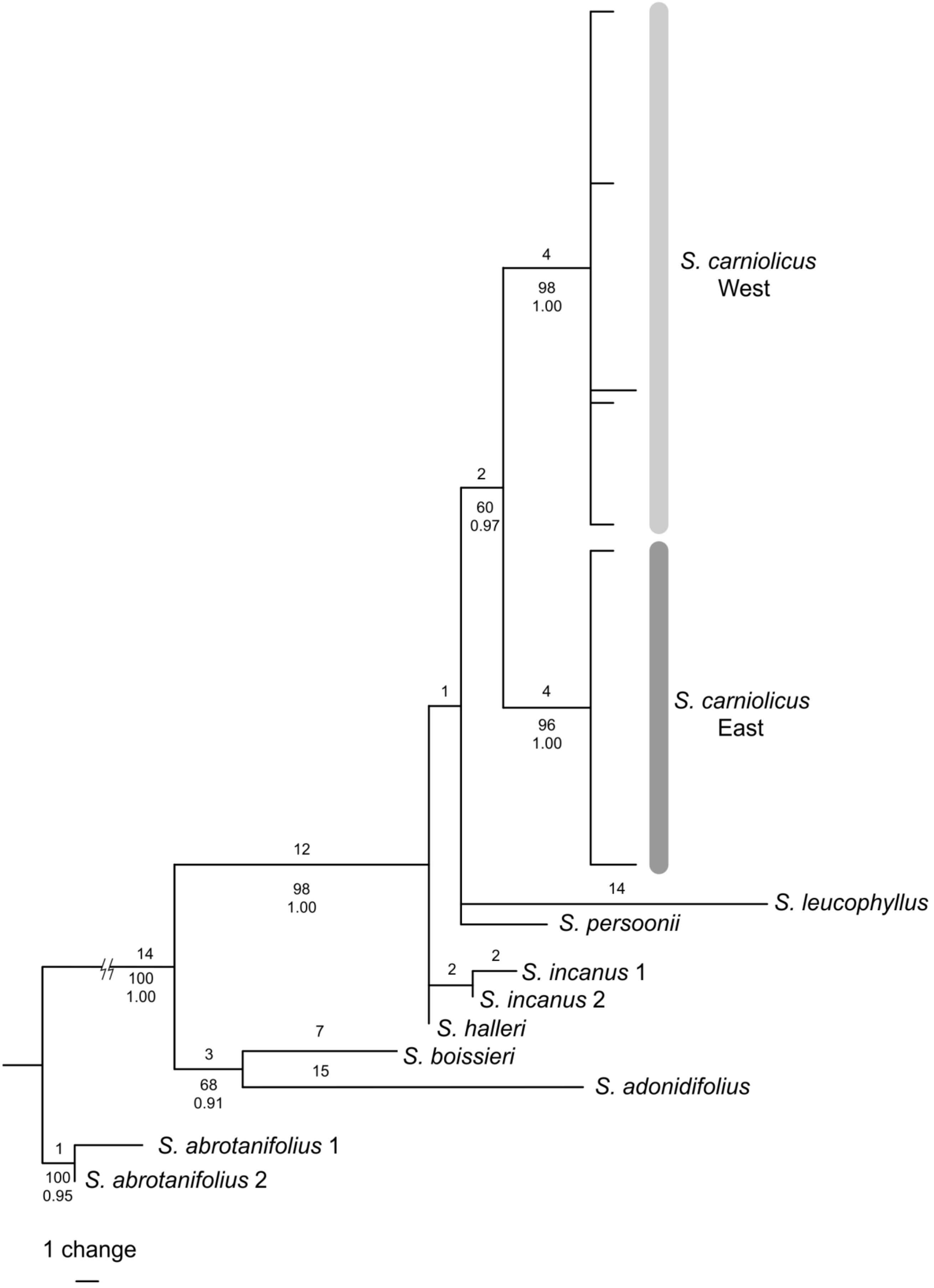

Fig. S2 Population dendrogram based on agglomerative hierarchical clustering using geographic distances. The two distance cut-offs (ca. 40 and ca. 70 km) and the corresponding circumscription of geographic regions (R1 to R13) are indicated. Population acronyms as in Table S1.

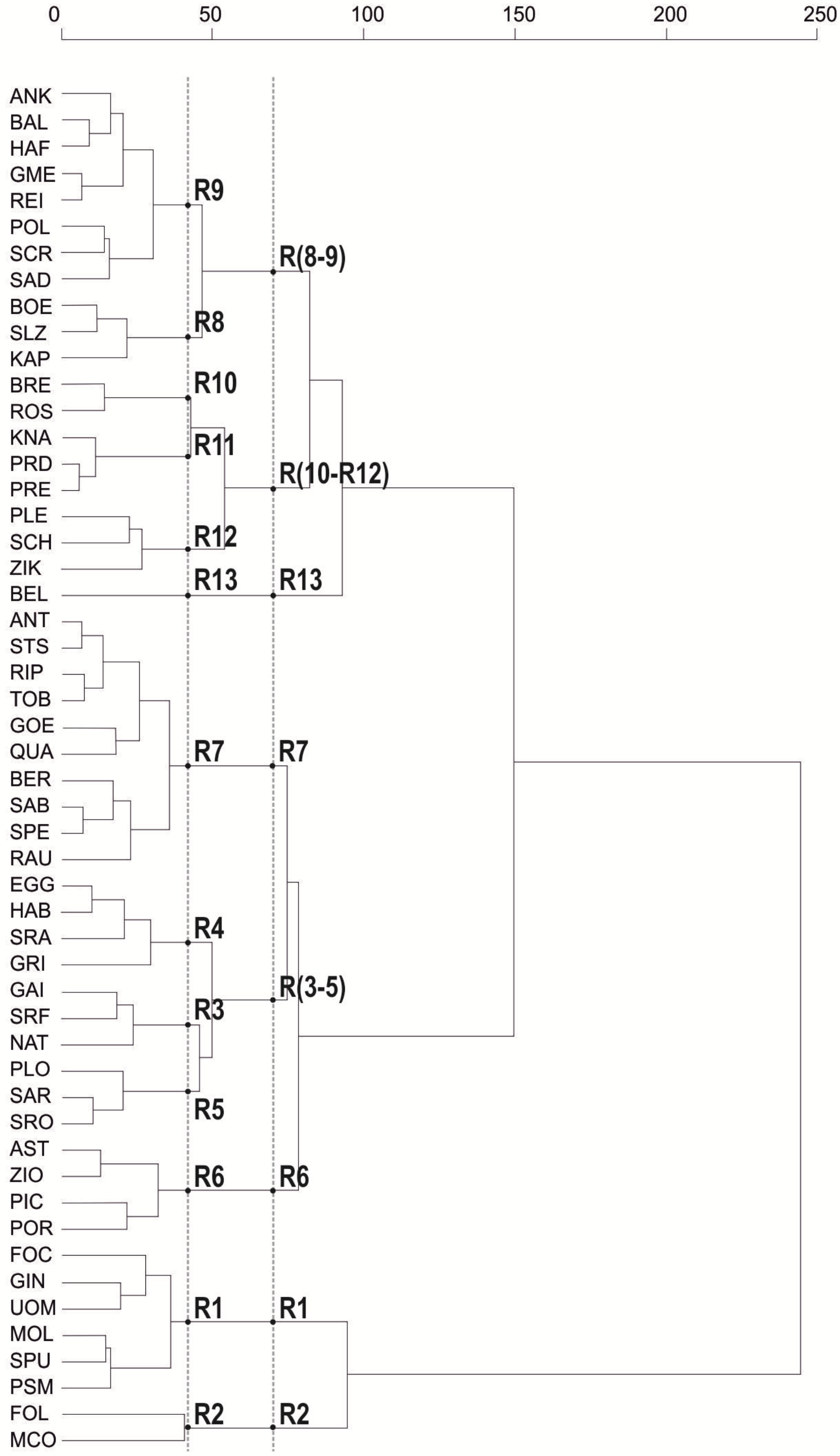

Fig. S3 Principal coordinate analysis (PCoA) of a matrix of pairwise Nei72 distances derived from AFLP analysis of diploid *Senecio carniolicus*.

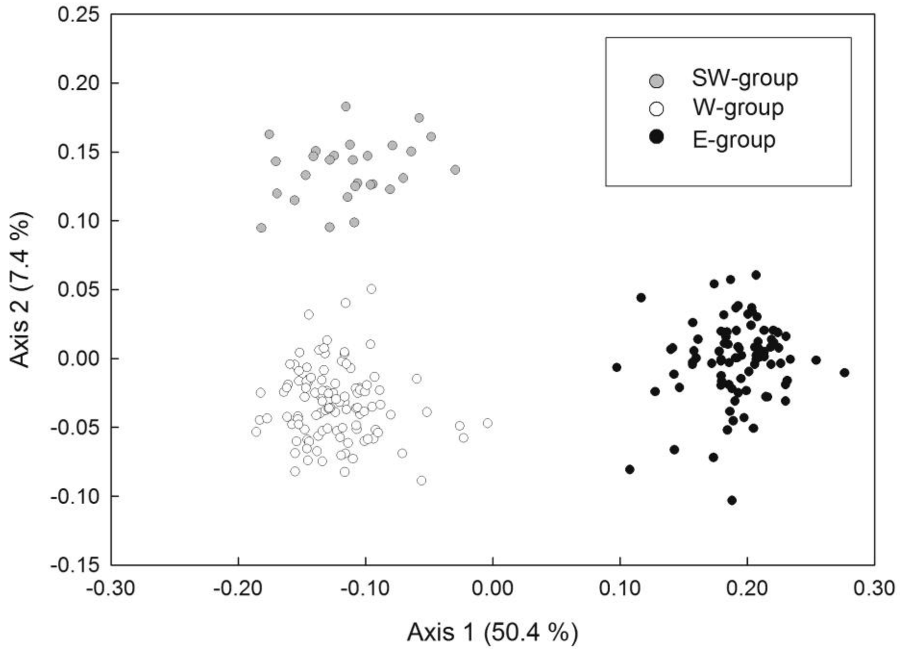

Fig. S4 Population Graphs network illustrating the genetic covariance structure among populations in diploid *Senecio carniolicus*. Populations are connected by edges if their genetic structures are conditionally dependent. Normal edges are those where the physical distances are proportional to the genetic distances. If they are not, then the populations are either closer (compressed edges) or further apart (extended edges) than expected given the genetic distances. Populations are numbered as in Fig. 1.

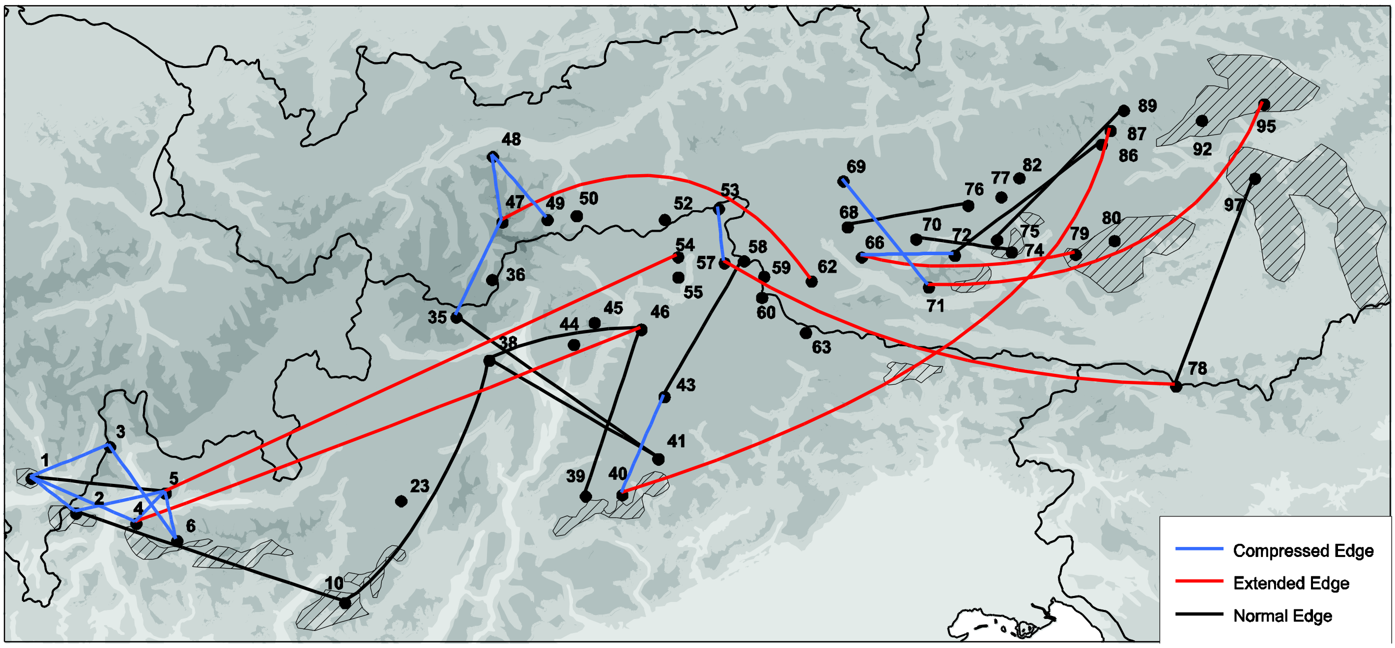

Fig. S5 Bayes Factor support for diffusion rates in a discrete non-reversible geographic diffusion model involving (a) 13 or (b) 8 discrete geographical regions. Source and sink regions are arranged on the x- and y-axis, respectively, Bayes Factors are shown on the z-axis. Prior means of the Poisson distribution are indicated beneath each panel (the default one in red); panels with means of 1 or more are shown twice to aid legibility (due to excessively high Bayes factors at mean 0.1).

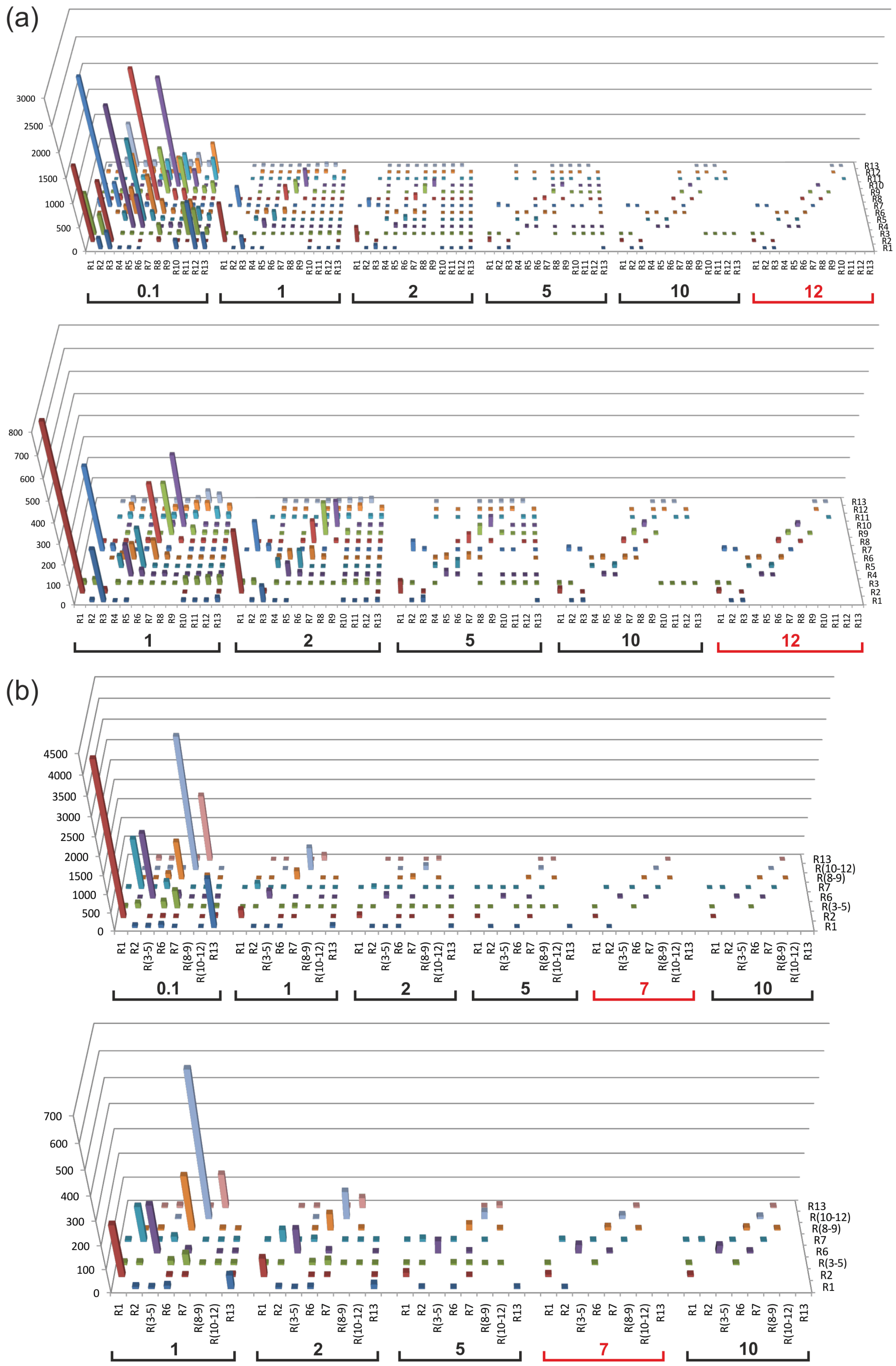

Table S1 Population numbers and acronyms, sampling locations and their coordinates, number of analyzed individuals, population diversity measures, ITS ribotypes, plastid haplotypes, and GenBank accession numbers for diploid *Senecio carniolicus*.

Population number[a]	Population acronym	Locality (coordinates)[b]	N_{AFLP}	DW[c]	ITS ribotype	N_{cpDNA}	Plastid haplotype[d]	Π[e]	GenBank accession numbers[f]
1	UOM	CH: Cima dell Uomo (N 46.23° E 8.94°)	4	0.99	1	4	H13	0.00	FR796910–FR796913; FR797128–FR797131; FR797346–FR797349; FR797564–FR797567; FR797782–FR797785
2	GIN	IT: Pizzo di Gino (N 46.12° E 9.14°)	4	1.00	1	4	$H12_2$, $H15_2$	1.33	FR796752–FR796755; FR796970–FR796973; FR797188–FR797191; FR797406–FR797409; FR797624–FR797627
3	FOC	IT: Campanile di Val Marina (N 46.33° E 9.29°)	4	1.00	1	4	$H12_2$, $H13_1$, $H18_1$	1.67	FR796737–FR796740; FR796957–FR796960; FR797175–FR797178; FR797393–FR797396; FR797611–FR797614
4	MOL	IT: Monte Legnone (N 46.09° E 9.42°)	4	1.25	1	4	H13	0.00	FR796790–FR796793; FR797007–FR797010; FR797225–FR797228; FR797443–FR797446; FR797661–FR797664
5	SPU	IT: Passo Locino (N 46.19° E 9.55°)	8	1.09	1	7	$H13_6$, $H18_1$	0.57	FR796884–FR796891; FR797103–FR797109; FR797321–FR797327; FR797539–FR797545; FR797757–FR797763
6	PSM	IT: Monte Verrobbio (N 46.04° E 9.60°)	4	1.29	1	4	H14	0.00	FR796828–FR796831; FR797046–FR797049; FR797264–FR797267; FR797482–FR797485; FR797700–FR797703
10	MCO	IT: Monte Colombine (N 45.85° E 10.36°)	4	1.26	1	4	$H2_3$, $H17_1$	4.00	FR796786–FR796789; FR797003–FR797006; FR797221–FR797224; FR797439–FR797442; FR797657–FR797660
23	FOL	IT: Cresta del Belvedere (N 46.16° E 10.62°)	7	1.03	1	5	$H13_1$, $H17_4$	0.40	FR796741–FR796747; FR796961–FR796965; FR797179–FR797183; FR797397–FR797401; FR797615–FR797619
35	SRF	IT: Schröfwand (N 46.74° E 10.87°)	2	1.02	1	2	H17	0.00	FR796896–FR796897; FR797114–FR797115; FR797332–FR797333; FR797550–FR797551; FR797768–FR797769
36	GAI	AT: Festkogel (N 46.86° E 11.04°)	4	0.93	1, 3	4	H35	0.00	FR796748–FR796751; FR796966–FR796969; FR797184–FR797187; FR797402–FR797405; FR797620–FR797623

38	NAT	IT: Naturnser Hochwart (N 46.6° E 11.02°)	4	1.08	1	4	$H19_1$, $H35_3$	1.00	FR796794–FR796797; FR797011–FR797014; FR797229–FR797232; FR797447–FR797450; FR797665–FR797668
39	ZIO	IT: Monte Ziolera (N 46.17° E 11.45°)	4	1.24	1	4	$H10_1$, $H35_3$	6.00	FR796918–FR796921; FR797136–FR797139; FR797354–FR797357; FR797572–FR797575; FR797790–FR797793
40	AST	IT: Cima D'Asta (N 46.17° E 11.62°)	2	1.27	1	2	$H9_1$, $H23_1$	10.00	FR796930–FR796931; FR797148–FR797149; FR797366–FR797367; FR797584–FR797585
41	PIC	IT: Cavallazza Piccola (N 46.28° E 11.78°)	4	1.46	1	4	$H1_1$, $H35_3$	4.50	FR796798–FR796801; FR797015–FR797018; FR797233–FR797236; FR797451–FR797454; FR797669–FR797672
43	POR	IT: Pre de Ciapel (N 46.48° E 11.82°)	4	0.96	1	4	H35	0.00	FR796814–FR796817; FR797031–FR797034; FR797249–FR797252; FR797467–FR797470; FR797685–FR797688
44	SAR	IT: Sarner Scharte (N 46.65° E 11.41°)	4	1.00	1	4	$H22_1$, $H35_3$	1.00	FR796860–FR796863; FR797082–FR797085; FR797300–FR797303; FR797518–FR797521; FR797736–FR797739
45	SRO	IT: Schrotthorn (N 46.72° E 11.51°)	4	1.11	1	4	$H22_1$, $H23_1$, $H35_2$	1.17	FR796898–FR796901; FR797116–FR797119; FR797334–FR797337; FR797552–FR797555; FR797770–FR797773
46	PLO	IT: Plose (N 46.69° E 11.72°)	4	1.07	1	4	$H9_1$, $H23_2$, $H35_1$	5.17	FR796806–FR796809; FR797023–FR797026; FR797241–FR797244; FR797459–FR797462; FR797677–FR797680
47	SRA	AT: Schrankogel (N 47.04° E 11.09°)	4	0.97	1	4	H35	0.00	FR796892–FR796895; FR797110–FR797113; FR797328–FR797331; FR797546–FR797549; FR797764–FR797767
48	GRI	AT: Rietzer Grießkogel (N 47.24° E 11.05°)	4	1.03	1	4	H35	0.00	FR796764–FR796767; FR796982–FR796985; FR797200–FR797203; FR797418–FR797421; FR797636–FR797639
49	HAB	AT: Habicht (N 47.04° E 11.30°)	4	0.90	1	4	$H23_2$, $H24_1$, $H35_1$	1.00	FR796768–FR796771; FR796986–FR796989; FR797204–FR797207; FR797422–FR797425; FR797640–FR797643
50	EGG	AT: Nößlachjoch, Eggersteller (N 47.05° E 11.43°)	4	1.32	1	4	$H34_2$, $H35_2$	0.67	FR796733–FR796736; FR796955–FR796956; FR797173–FR797174; FR797391–FR797392; FR797609–FR797610
52	BER	AT: Saurüssel (N 47.03° E 11.84°)	4	1.21	1	4	$H1_2$, $H13_1$, $H16_1$	6.50	FR796721–FR796724; FR796943–FR796946; FR797161–FR797164; FR797379–FR797382; FR797597–FR797600

53	RAU	IT: Rauchkofel (N 47.06° E 12.09°)	4	1.10	1	4	$H11_1$, $H13_1$, $H28_1$, $H35_1$	6.17	FR796836–FR796839; FR797054–FR797057; FR797272–FR797275; FR797490–FR797493; FR797708–FR797711
54	SPE	IT: Speikboden (N 46.92° E 11.90°)	4	1.06	1	4	$H3_1$, $H11_1$, $H35_2$	8.33	FR796880–FR796883; FR797099–FR797102; FR797317–FR797320; FR797535–FR797538; FR797753–FR797756
55	SAB	IT: Sambock (N 46.85° E 11.89°)	4	1.00	1	4	$H5_2$, $H35_2$	7.33	FR796852–FR796855; FR797074–FR797077; FR797292–FR797295; FR797510–FR797513; FR797728–FR797731
57	ANT	IT: Antholzer Scharte (N 46.89° E 12.11°)	4	1.38	1	4	$H11_2$, $H33_1$, $H35_1$	7.17	FR796705–FR796708; FR796926–FR796929; FR797144–FR797147; FR797362–FR797365; FR797580–FR797583
58	STS	AT: Almerhorn (N 46.90° E 12.20°)	4	1.36	1	4	$H4_1$, $H20_1$, $H21_1$, $H35_1$	6.50	FR796902–FR796905; FR797120–FR797123; FR797338–FR797341; FR797556–FR797559; FR797774–FR797777
59	RIP	IT: Riepenspitz (N 46.85° E 12.29°)	4	1.08	1	4	$H11_1$, $H34_1$, $H35_2$	4.50	FR796844–FR796847; FR797066–FR797069; FR797284–FR797287; FR797502–FR797505; FR797720–FR797723
60	TOB	IT: Toblacher Pfannhorn (N 46.78° E 12.28°)	4	1.24	1	4	H35	0.00	FR796906–FR796909; FR797124–FR797127; FR797342–FR797345; FR797560–FR797563; FR797778–FR797781
62	GOE	AT: Gölbner (N 46.83° E 12.51°)	4	0.92	1	4	$H1_2$, $H35_2$	6.00	FR796760–FR796763; FR796978–FR796981; FR797196–FR797199; FR797414–FR797417; FR797632–FR797635
63	QUA	IT: Col Quaternà (N 46.67° E 12.47°)	4	1.17	1	4	$H1_1$, $H3_1$, $H35_2$	7.17	FR796832–FR796835; FR797050–FR797053; FR797268–FR797271; FR797486–FR797489; FR797704–FR797707
66	SLZ	AT: Schleinitz (N 46.90° E 12.74°)	4	1.36	7	4	$H3_1$, $H31_2$, $H35_1$	5.67	FR796876–FR796879; FR797095–FR797098; FR797313–FR797316; FR797531–FR797534; FR797749–FR797752
68	BOE	AT: Schönleitenspitze (N 46.99° E 12.68°)	4	1.37	7	4	$H31_2$, $H35_1$	0.67	FR796725–FR796728; FR796947–FR796950; FR797165–FR797168; FR797383–FR797386; FR797601–FR797604
69	KAP	AT: Kapruner Törl (N 47.14° E 12.67°)	6	1.41	4, 5, 7, 8, 9, 10	5	$H25_1$, $H26_1$, $H35_3$	0.80	FR796776–FR796781; FR796994–FR796998; FR797212–FR797216; FR797430–FR797434; FR797648–FR797652

70	SAD	AT: Sadnig (N 46.95° E 12.99°)	4	1.00	7	4	$H31_1$, $H35_3$	0.50	FR796856–FR796859; FR797078–FR797081; FR797296–FR797299; FR797514–FR797517; FR797732–FR797735
71	SCR	AT: Scharnik (N 46.79° E 13.04°)	4	1.16	7	4	$H31_3$, $H35_1$	0.50	FR796872–FR796875; FR797091–FR797094; FR797309–FR797312; FR797527–FR797530; FR797745–FR797748
72	POL	AT: Polinik (N 46.89° E 13.17°)	4	1.18	1, 7	4	$H26_1$, $H35_3$	0.50	FR796810–FR796813; FR797027–FR797030; FR797245–FR797248; FR797463–FR797466; FR797681–FR797684
74	GME	AT: Gmeineck (N 46.89° E 13.43°)	4	1.12	7	4	$H29_2$, $H35_2$	0.67	FR796756–FR796759; FR796974–FR796977; FR797192–FR797195; FR797410–FR797413; FR797628–FR797631
75	REI	AT: Reißeck (N 46.93° E 13.36°)	8	1.43	7	8	$H6_1$, $H7_2$, $H8_2$, $H35_3$	4.75	FR796840–FR796843; FR797058–FR797065; FR797276–FR797283; FR797494–FR797501; FR797712–FR797719
76	ANK	AT: Ankogel (N 47.05° E 13.24°)	4	1.20	7	4	H35	0.00	FR796701–FR796704; FR796922–FR796925; FR797140–FR797143; FR797358–FR797361; FR797576–FR797579
77	HAF	AT: Großer Hafner (N 47.07° E 13.39°)	4	1.04	7	4	H35	0.00	FR796772–FR796775; FR796990–FR796993; FR797208–FR797211; FR797426–FR797429; FR797644–FR797647
78	BEL	AT / SLO: Belščica (N 46.44° E 14.15°)	8	1.16	1, 2, 6, 7	7	H35	0.00	FR796713–FR796720; FR796936–FR796942; FR797154–FR797160; FR797372–FR797378; FR797590–FR797596
79	ROS	AT: Rosennock (N 46.87° E 13.72°)	4	1.25	7	4	$H27_1$, $H32_1$, $H35_2$	1.00	FR796848–FR796851; FR797070–FR797073; FR797288–FR797291; FR797506–FR797509; FR797724–FR797727
80	BRE	AT: Bretthöhe (N 46.91° E 13.90°)	4	1.05	7	4	H35	0.00	FR796729–FR796732; FR796951–FR796954; FR797169–FR797172; FR797387–FR797390; FR797605–FR797608
82	BAL	AT: Balonspitze (N 47.12° E 13.48°)	4	1.01	7	4	$H30_1$, $H31_1$, $H32_1$, $H35_1$	1.50	FR796709–FR796712; FR796932–FR796935; FR797150–FR797153; FR797368–FR797371; FR797586–FR797589
86	PRE	AT: Preber (N 47.22° E 13.87°)	4	1.14	7	4	H35	0.00	FR796824–FR796827; FR797042–FR797045; FR797260–FR797263; FR797478–FR797481; FR797696–FR797699

87	PRD	AT: Predigstuhl (N 47.26° E 13.91°)	8	1.23	7	7	H35	0.00	FR796818–FR796823; FR797035–FR797041; FR797253–FR797259; FR797471–FR797477; FR797689–FR797695
89	KNA	AT: Großer Knallstein (N 47.32° E 13.98°)	4	1.57	7	4	$H31_1$, $H35_3$	0.50	FR796782–FR796785; FR796999–FR797002; FR797217–FR797220; FR797435–FR797438; FR797653–FR797656
92	SCH	AT: Schießeck (N 47.27° E 14.34°)	6	1.32	7	5	H35	0.00	FR796864–FR796871; FR797086–FR797090; FR797304–FR797308; FR797522–FR797526; FR797740–FR797744
95	PLE	AT: Großer Ringkogel (N 47.31° E 14.63°)	4	1.09	7	4	H35	0.00	FR796802–FR796805; FR797019–FR797022; FR797237–FR797240; FR797455–FR797458; FR797673–FR797676
97	ZIK	AT: Zirbitzkogel (N 47.08° E 14.57°)	4	1.19	7	4	H35	0.00	FR796914–FR796917; FR797132–FR797135; FR797350–FR797353; FR797568–FR797571; FR797786–FR797789

[a]population numbers as in Sonnleitner *et al.* (2010)

[b]AT: Austria; CH: Switzerland; I: Italy; SLO: Slovenia

[c]within-population rarity of markers as frequency-down-weighted marker values calculated according to Schönswetter & Tribsch (2005)

[d]haplotype numbers as in Fig. 3; in case of intrapopulational polymorphism, the number of individuals with a given haplotype is indicated as subscript numbers

[e]number of pairwise differences among haplotypes within populations (Tajima 1983)

[f]nuclear ITS; plastid *pet*L–*psb*E spacer; plastid *rpl*32–*trn*L spacer; plastid *psb*D–*trn*T spacer; plastid *rps*16–*trn*K spacer

Table S2 Sampling localities and their geographic coordinates as well as GenBank accession numbers of four plastid regions for outgroup species of the *Incani* clade.

Taxon	Locality (coordinates)[a]	GenBank accession numbers[b]
Senecio abrotanifolius L. 1	I: Lombardia, Sondrio (N 46°11'40" E 09°33'50")	HE614512; HE614440; HE614296; HE614368
Senecio abrotanifolius L. 2	I: Südtirol, Paso Sella (N 46°30'30" E 11°46'00")	HE614513; HE614441; HE614297; HE614369
Senecio adonidifolius Loisel. 1	F: Pyrénées-Orientales, Col de Mantet (N 42°29'00" E 02°19'00")	HE614514; HE614442; HE614298; HE614370
Senecio adonidifolius Loisel. 2	F: Pyrénées-Orientales, Col de Mantet (N 42°29'00" E 02°19'00")	HE614515; HE614443; HE614299; HE614371
Senecio boissieri DC. 1	E: Granada, Pico del Tosal (N 37°02'01" E 03°24'46")	HE614516; HE614444; HE614300; HE614372
Senecio boissieri DC. 2	E: Granada, Mulhacén (N 37°03'12" E 03°18'42")	HE614517; HE614445; HE614301; HE614373
Senecio halleri Dandy 1	F: Rhône-Alpes, Col des Evettes (N 45°35'00" E 07°06'00")	HE614518; HE614446; HE614302; HE614374
Senecio halleri Dandy 2	F: Rhône-Alpes, Col des Evettes (N 45°35'00" E 07°06'00")	HE614519; HE614447; HE614303; HE614375
Senecio halleri Dandy 3	I: Piemonte, Rifugio Vittorio Emanuele II (N 45°39'00" E 07°16'00")	HE614522; HE614450; HE614306; HE614378
Senecio halleri Dandy 4	I: Piemonte, Rifugio Vittorio Emanuele II (N 45°39'00" E 07°16'00")	HE614525; HE614453; HE614309; HE614381
Senecio halleri Dandy 5	I: Aosta, Le refuge Arp (N 45°46'00" E 07°45'00")	HE614520; HE614448; HE614304; HE614376
Senecio halleri Dandy 6	I: Aosta, Le refuge Arp (N 45°46'00" E 07°45'00")	HE614521; HE614449; HE614305; HE614377
Senecio halleri Dandy 7	CH: Valais, Gornergrat (N 45°59'00" E 07°48'00")	HE614530; HE614458; HE614314; HE614386
Senecio halleri Dandy 8	CH: Valais, Gornergrat (N 45°59'00" E 07°48'00")	HE614531; HE614459; HE614315; HE614387
Senecio halleri Dandy 9	I: Piemonte, Ceppo Morelli (N 45°59'00" E 08°02'00")	HE614523; HE614451; HE614307; HE614379
Senecio halleri Dandy 10	I: Piemonte, Ceppo Morelli (N 45°59'00" E 08°02'00")	HE614524; HE614452; HE614308; HE614380
Senecio halleri Dandy 11	I: Piemonte, Monte Zeda (N 46°03'00" E 08°32'00")	HE614526; HE614454; HE614310; HE614382
Senecio halleri Dandy 12	I: Piemonte, Monte Zeda (N 46°03'00" E 08°32'00")	HE614527; HE614455; HE614311; HE614383
Senecio halleri Dandy 13	CH: Valais, Seehorn (N 46°10'00" E 08°07'00")	HE614532; HE614460; HE614316; HE614388
Senecio halleri Dandy 14	CH: Valais, Breithorn (N 46°18'00" E 08°05'00")	HE614528; HE614456; HE614312; HE614384
Senecio halleri Dandy 15	CH: Valais, Breithorn (N 46°18'00" E 08°05'00")	HE614529; HE614457; HE614313; HE614385
Senecio incanus L. 1	F: Alpes-Maritimes, N Col de la Lombarde (N 44°12'10" E 07°08'55")	HE614541; HE614469; HE614325; HE614397
Senecio incanus L. 2	F: Alpes-Maritimes, N Col de la Lombarde (N 44°12'10" E 07°08'55")	HE614542; HE614470; HE614326; HE614398

Senecio incanus L. 3	F: Alpes-Maritimes, Cime des Trois Serrières (N 44°20'30" E 06°50'45")	HE614545; HE614473; HE614329; HE614401
Senecio incanus L. 4	F: Alpes-Maritimes, Cime des Trois Serrières (N 44°20'30" E 06°50'45")	HE614546; HE614474; HE614330; HE614402
Senecio incanus L. 5	F: Hautes-Alpes, Col de Vars (N 44°32'25" E 06°42'15")	HE614547; HE614475; HE614331; HE614403
Senecio incanus L. 6	F: Hautes-Alpes, Col de Vars (N 44°32'25" E 06°42'15")	HE614548; HE614476; HE614332; HE614404
Senecio incanus L. 7	F: Hautes-Alpes, Col Agnel - Le Pain de Sucre (N 44°41'05" E 06°58'50")	HE614535; HE614463; HE614319; HE614391
Senecio incanus L. 8	F: Hautes-Alpes, Col Agnel - Le Pain de Sucre (N 44°41'05" E 06°58'50")	HE614536; HE614464; HE614320; HE614392
Senecio incanus L. 9	I: Piemonte, Punta Cialancia (N 44°52'35" E 07°07'20")	HE614539; HE614467; HE614323; HE614395
Senecio incanus L. 10	I: Piemonte, Punta Cialancia (N 44°52'35" E 07°07'20")	HE614540; HE614468; HE614324; HE614396
Senecio incanus L. 11	F: Hautes-Alpes, Colle Basset (N Sestrière) (N 44°59'45" E 06°52'30")	HE614543; HE614471; HE614327; HE614399
Senecio incanus L. 12	F: Hautes-Alpes, Colle Basset (N Sestrière) (N 44°59'45" E 06°52'30")	HE614544; HE614472; HE614328; HE614400
Senecio incanus L. 13	F: Hautes-Alpes, Col de Laurichard (N 45°06'20" E 06°24'00")	HE614561; HE614489; HE614345; HE614417
Senecio incanus L. 14	F: Hautes-Alpes, Col de Laurichard (N 45°06'20" E 06°24'00")	HE614562; HE614490; HE614346; HE614418
Senecio incanus L. 15	I: Piemonte, Colle Sommeiller (N 45°07'50" E 06°50'10")	HE614565; HE614493; HE614349; HE614421
Senecio incanus L. 16	I: Piemonte, Colle Sommeiller (N 45°07'50" E 06°50'10")	HE614566; HE614494; HE614350; HE614422
Senecio incanus L. 17	I: Piemonte, Monte Palon (N 45°12'30" E 07°08'40")	HE614563; HE614491; HE614347; HE614419
Senecio incanus L. 18	I: Piemonte, Monte Palon (N 45°12'30" E 07°08'40")	HE614564; HE614492; HE614348; HE614420
Senecio incanus L. 19	F: Savoie, Col de l'Iseran (N 45°25'05" E 07°01'55")	HE614549; HE614477; HE614333; HE614405
Senecio incanus L. 20	F: Savoie, Col de l'Iseran (N 45°25'05" E 07°01'55")	HE614550; HE614478; HE614334; HE614406
Senecio incanus L. 21	I: Piemonte, Grajische Alpen (N 45°37'56" E 07°14'24")	HE614557; HE614485; HE614341; HE614413
Senecio incanus L. 22	F: Savoie, Petit St. Bernhard (N 45°40'20" E 06°52'40")	HE614551; HE614479; HE614335; HE614407
Senecio incanus L. 23	F: Savoie, Petit St. Bernhard (N 45°40'20" E 06°52'40")	HE614552; HE614480; HE614336; HE614408
Senecio incanus L. 24	F: Savoie, Petit St. Bernhard (N 45°40'20" E 06°52'40")	HE614558; HE614486; HE614342; HE614414
Senecio incanus L. 25	CH: Valais, Grand St. Bernhard (N 45°52'50" E 07°10'12")	HE614555; HE614483; HE614339; HE614411
Senecio incanus L. 26	CH: Valais, Grand St. Bernhard (N 45°52'50" E 07°10'12")	HE614556; HE614484; HE614340; HE614412
Senecio incanus L. 27	CH: Valais, Grosser St. Bernhard-Pass (N 45°55'30" E 07°15'14")	HE614577; HE614505; HE614361; HE614433
Senecio incanus L. 28	CH: Valais, Grosser St. Bernhard-Pass (N 45°55'30" E 07°15'14")	HE614578; HE614506; HE614362; HE614434
Senecio incanus L. 29	CH: Valais, Grosser St. Bernhard-Pass (N 45°55'30" E 07°15'14")	HE614579; HE614507; HE614363; HE614435

Senecio incanus L. 30	CH: Valais, Grosser St. Bernhard-Pass (N 45°55'30" E 07°15'14")	HE614580; HE614508; HE614364; HE614436
Senecio incanus L. 31	F: Haute-Savoie, Le Brevent (N 45°56'10" E 06°51'00")	HE614574; HE614502; HE614358; HE614430
Senecio incanus L. 32	F: Haute-Savoie, Le Brevent (N 45°56'10" E 06°51'00")	HE614575; HE614503; HE614359; HE614431
Senecio incanus L.33	CH: Valais, Gornergrat (N 45°59'00" E 07°48'00")	HE614537; HE614465; HE614321; HE614393
Senecio incanus L.34	CH: Valais, Gornergrat (N 45°59'00" E 07°48'00")	HE614538; HE614466; HE614322; HE614394
Senecio incanus L. 35	CH: Valais, Rifelhorn (N 45°59'00" E 07°48'00")	HE614553; HE614481; HE614337; HE614409
Senecio incanus L. 36	CH: Valais, Rifelhorn (N 45°59'00" E 07°48'00")	HE614554; HE614482; HE614338; HE614410
Senecio incanus L. 37	CH: Valais, Montagne d'Arolla (N 46°00'56" E 07°26'14")	HE614571; HE614499; HE614355; HE614427
Senecio incanus L. 38	CH: Valais, Montagne d'Arolla (N 46°00'56" E 07°26'14")	HE614572; HE614500; HE614356; HE614428
Senecio incanus L. 39	CH: Valais, Montagne d'Arolla (N 46°00'56" E 07°26'14")	HE614573; HE614501; HE614357; HE614429
Senecio incanus L. 40	CH: Valais, Simplon (N 46°15'28" E 08°04'51")	HE614576; HE614504; HE614360; HE614432
Senecio incanus L. 41	CH: Ticino, Nufenenstock (N 46°28'45" E 08°23'15")	HE614533; HE614461; HE614317; HE614389
Senecio incanus L. 42	CH: Ticino, Nufenenstock (N 46°28'45" E 08°23'15")	HE614534; HE614462; HE614318; HE614390
Senecio incanus L. 43	CH: Ticino, Nufenenstock (N 46°28'45" E 08°23'15")	HE614559; HE614487; HE614343; HE614415
Senecio incanus L. 44	CH: Ticino, Nufenenstock (N 46°28'45" E 08°23'15")	HE614560; HE614488; HE614344; HE614416
Senecio leucophyllus DC. 1	F: Pyrénées-Orientales, Puigmal d'Err (N 42°22'59" E 02°07'01")	HE614582; HE614510; HE614366; HE614438
Senecio leucophyllus DC. 2	F: Pyrénées-Orientales, Petit Puigmal de Sègre (N 42°23'00" E 02°07'00")	HE614567; HE614495; HE614351; HE614423
Senecio leucophyllus DC. 3	F: Pyrénées-Orientales, Petit Puigmal de Sègre (N 42°23'00" E 02°07'00")	HE614568; HE614496; HE614352; HE614424
Senecio leucophyllus DC. 4	F: Pyrénées-Orientales, Pic du Canigou (N 42°30'30" E 02°26'00")	HE614583; HE614511; HE614367; HE614439
Senecio leucophyllus DC. 5	F: Ardèche, Mont Mézenc (N 44°54'48" E 04°11'27")	HE614581; HE614509; HE614365; HE614437
Senecio persoonii De Not. 1	I: Piemonte, Viozene (N 44°08'45" E 07°47'05")	HE614569; HE614497; HE614353; HE614425
Senecio persoonii De Not. 2	I: Piemonte, Viozene (N 44°08'45" E 07°47'05")	HE614570; HE614498; HE614354; HE614426

[b]CH: Switzerland; E: Spain; F: France; I: Italy

[f]nuclear ITS; plastid *pet*L–*psb*E spacer; plastid *rpl*32–*trn*L spacer; plastid *psb*D–*trn*T spacer; plastid *rps*16–*trn*K spacer

Table S3 Newly designed internal primers for amplification of three plastid spacer regions.

Region	Primer	
*psb*D–*trn*T	psbD-F2	5'–GTACATCAAGCTGCGGTCTG–3'
	trnT-R2	5'–GGTAGAGCACTCCTATGGTAAGG–3'
*rps*16–*trn*K	rps16-F2	5'–ATATGCATATGCAGTGCCAATCC–3'
	trnK-R2	5'–CTCTACCGTTGAGTTAGCAACCC–3'
*psb*E–*pet*L	psbE-F2	5'–CATATTCTTGTACAGTCAAAAGG–3'
	petL-R2	5'–TATGTTCTTTTTATACATATGTTTATAA–3'

Table S4 Sequence statistics.

Region	Sequence length[a]	Alignment length	Gaps	Informative positions (with/without outgroups)
ITS	865–866	866	1	10/17
*psb*D—*trn*T	670–681	672	5	4/3
*rps*16—*trn*K	590–602	594	3	5/3
*psb*E—*pet*L	1175–1199	1181	8	14/9
*trn*L—*rpl*32	641–946	931	2	15/12
combined plastid regions	—	3378	18	38/27

[a]for samples where some regions needed to be amplified with interior primers the missing sequence stretches were assumed to be of equal length as in the majority of samples

Table S5 Bayes Factor support for geographic diffusion rates under the reversible discrete model using (a) 13 regions (R1–R13) or (b) 8 regions (regions comprising more than one of the 13 regions distinguished in (a) have composite names) with different prior means for the truncated Poisson distribution (the row with the default value is boxed) as well as the ratio of the maximum (indicated in bold) to the minimum Bayes Factor support (indicated in italics) for a given rate (see text for details). Those connections, which have rates supported by Bayes Factors of at least 3 with the default mean (*ln*2 = 0.693) for the truncated Poisson prior (see text for details), are shown left of the grey bar and have the smallest and largest Bayes Factors indicated in italics and bold, respectively, and Bayes Factors below 3 are indicated by underlining. Connections, which are supported only under higher means for the truncated Poisson prior, are shown to the right of the grey bar.

(a)

mean	R1-R2	R1-R3	R2-R3	R3-R7	R4-R5	R5-R6	R6-R7	R6-R8	R7-R8	R8-R9	R9-R10		R2-R7	R4-R6	R8-R11	R9-R11	R11-R12
0.1	*6.64*	*4.11*	**5.84**	**5.91**	*16.19*	*21.41*	*4.37*	**4.13**	*11.75*	*19.88*	14.93						
0.693	8.03	4.99	5.41	4.53	17.97	24.91	6.05	3.43	12.26	20.71	*14.62*						
1	8.56	4.91	5.10	3.82	18.62	28.84	6.60	3.20	14.02	29.76	16.25		3.76				
2	9.04	6.18	4.86	3.38	20.57	33.61	8.78	*2.84*	15.73	29.60	16.64		4.35				
5	11.77	7.13	4.56	*2.76*	22.73	42.67	12.11	2.94	17.72	31.81	20.97		6.17	3.47			
10	**15.41**	**9.47**	*4.50*	3.00	**27.27**	**56.71**	**17.31**	3.31	**23.00**	**35.04**	**31.81**		7.77	4.31	3.02	3.12	3.23
max/min	2.32	2.30	1.30	2.14	1.68	2.65	3.96	1.45	1.96	1.76	2.18						

(b)

mean	R1-R2	R2-R7	R(3-5)-R6	R6-R7	R7-R(8-9)	R(8-9)-R(10-12)
0.1	5.15	3.89	*135.67*	*3.67*	*15.24*	**118.24**
0.693	5.32	4.52	181.70	5.81	21.02	77.05
1	5.15	**4.59**	191.89	6.88	24.60	75.36
2	**5.71**	4.23	221.42	7.16	25.05	53.81
5	5.11	3.86	**239.27**	**8.92**	**28.74**	37.50
10	*4.30*	*3.15*	199.30	8.50	23.81	*22.99*
max/min	1.33	1.46	1.76	2.43	1.89	5.14

Table S6 Bayes Factor support for geographic diffusion rates under the non-reversible discrete model using (a) 13 regions (R1–R13) or (b) 8 regions (regions comprising more than one of the 13 regions distinguished in (a) have composite names) with different prior means for the truncated Poisson distribution (the row with the default value is boxed) as well as the ratio of the maximum (indicated in bold) to the minimum Bayes Factor support (indicated in italics) for a given rate (see text for details).

(a)

mean	R1→R2	R1→R3	R2→R1	R2→R3	R2→R7	R3→R1	R3→R2	R3→R7	R4→R5	R4→R6	R5→R4	R5→R6
0.1	**1559.39**	**865.66**	**227.96**	**417.71**	**2579.39**	**360.58**	**1250.05**	**494.75**	**168.29**	**164.42**	**2431.24**	**534.74**
1	797.64	18.56	11.15	28.71	411.02	259.17	21.72	29.31	59.96	29.57	156.26	77.85
2	305.59	15.95	8.73	21.65	145.53	79.31	12.42	27.97	39.79	15.83	64.94	42.96
5	62.10	12.05	5.56	7.46	48.41	22.47	9.16	10.33	15.38	8.37	27.47	19.74
10	24.97	8.23	3.85	5.00	18.30	7.19	5.14	7.01	8.91	5.39	11.85	14.26
12	*16.80*	*7.65*	*3.23*	*3.84*	*14.72*	*5.44*	*4.36*	*5.52*	*7.39*	*4.65*	*9.32*	*11.94*
max/min	92.82	113.16	70.58	108.78	175.23	66.28	286.71	89.63	22.77	35.36	260.86	44.79

mean	R6→R4	R6→R5	R7→R6	R7→R8	R8→R7	R8→R9	R9→R8	R9→R10	R10→R9	R11→R12	R11→R13	R12→R11	R12→R13
0.1	**667.25**	**1651.98**	**791.19**	**2602.09**	**50.36**	**927.04**	**200.37**	**2183.40**	**720.60**	**270.99**	**259.40**	41.96	**90.71**
1	32.94	195.27	96.31	290.18	14.84	252.93	18.50	350.19	36.95	40.29	58.38	**50.39**	42.66
2	21.15	86.41	56.64	108.89	7.74	158.15	12.21	125.45	19.55	27.47	16.55	13.89	20.86
5	8.57	39.09	26.51	43.68	6.59	45.58	9.07	49.54	11.15	9.64	7.79	7.76	8.02
10	4.53	17.48	14.49	17.44	5.26	20.03	6.41	22.44	6.39	4.31	4.11	3.77	3.72
12	*3.65*	*14.46*	*11.90*	*15.24*	*4.20*	*14.13*	*5.94*	*18.22*	*5.41*	*3.40*	*3.04*	*3.11*	*3.04*
max/min	182.81	114.24	66.49	170.74	11.99	65.61	33.73	119.84	133.20	79.70	85.33	16.20	29.84

(b)

mean	R1→R2	R1→R(3-5)	R1→R7	R2→R7	R(3-5) →R6	R6→R(3-5)	R6→R7	R7→R6	R7→R(8-9)	R(8-9)→R7	R(8-9)→R(10-12)	R(10-12)→R(8-9)	R(10-12)→R13
0.1	**4069.93**	**46.28**	**30.00**	**1342.30**	**1744.76**	**189.88**	**199.35**	**144.60**	**1013.76**	**18.41**	**3458.62**	**88.40**	**1730.45**
1	219.77	10.25	6.49	148.97	203.69	19.76	23.18	17.80	229.04	3.10	600.64	6.48	141.78
2	79.70	6.56	5.53	51.13	102.65	12.14	11.93	12.69	70.26	4.58	116.51	7.71	42.42
5	19.03	5.69	4.09	13.05	53.19	6.95	6.46	8.19	25.77	3.34	30.96	5.61	11.24
7	11.10	*4.07*	*3.00*	7.74	36.91	*5.17*	3.96	*6.56*	14.48	*3.04*	16.20	*4.63*	5.35
10	*8.70*	4.33	3.21	*5.93*	*33.45*	5.68	*3.47*	7.33	*12.12*	3.23	*10.26*	4.87	*4.19*
max/min	467.81	11.37	10.00	226.36	52.16	36.73	57.45	22.04	83.64	6.06	337.10	19.09	413.00
